# Supplementary material for: Global, regional, and national burden of early-onset OA attributable to high BMI: 1990–2021 estimates and 2036 projections from the global burden of disease study
Source: PLoS One. 2025 Jul 16;20(7):e0328414. doi: 10.1371/journal.pone.0328414 (PMC12266449; doi:10.1371/journal.pone.0328414)
Supplement: S1 Appendix — (DOCX) [file pone.0328414.s001.docx]

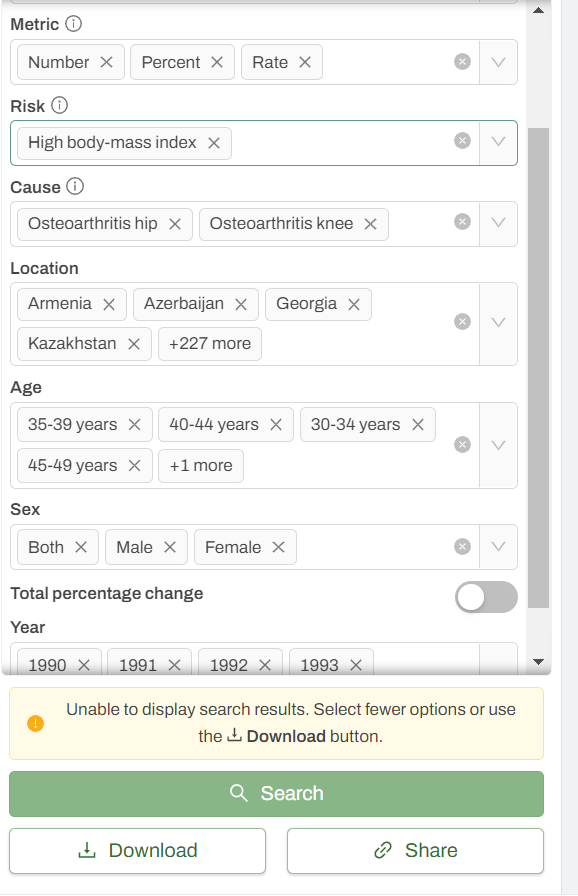


Appendix S1. Overview of the data screening of early-onset osteoarthritis attributable to high BMI in the 2021 Global Burden of Disease study
