# Supplementary material for: Global, regional, and national burden of early-onset OA attributable to high BMI: 1990–2021 estimates and 2036 projections from the global burden of disease study
Source: PLoS One. 2025 Jul 16;20(7):e0328414. doi: 10.1371/journal.pone.0328414 (PMC12266449; doi:10.1371/journal.pone.0328414)
Supplement: S2 Fig — Note: A, The ASDR of early-onset knee osteoarthritis attributable to high BMI among female in 1990, by country; B, The ASDR of early-onset knee osteoarthritis attributable to high among female BMI in 2021, by country; C, The trend in ASDR of early-onset knee osteoarthritis attributable to high BMI among female from 1990 to 2021; D, The ASDR of early-onset hip osteoarthritis attributable to high BMI among female in 1990, by country; E, The ASDR of early-onset hip osteoarthritis attributable to high BMI among female in 2021, by country; F, The trend in ASDR of early-onset hip osteoarthritis attributable to high BMI among female from 1990 to 2021. Abbreviations: BMI, Body mass index; EAPC, estimated annual percentage change; ASDR, age-standardized disability-adjusted life years rate. (DOCX) [file pone.0328414.s003.docx]

**
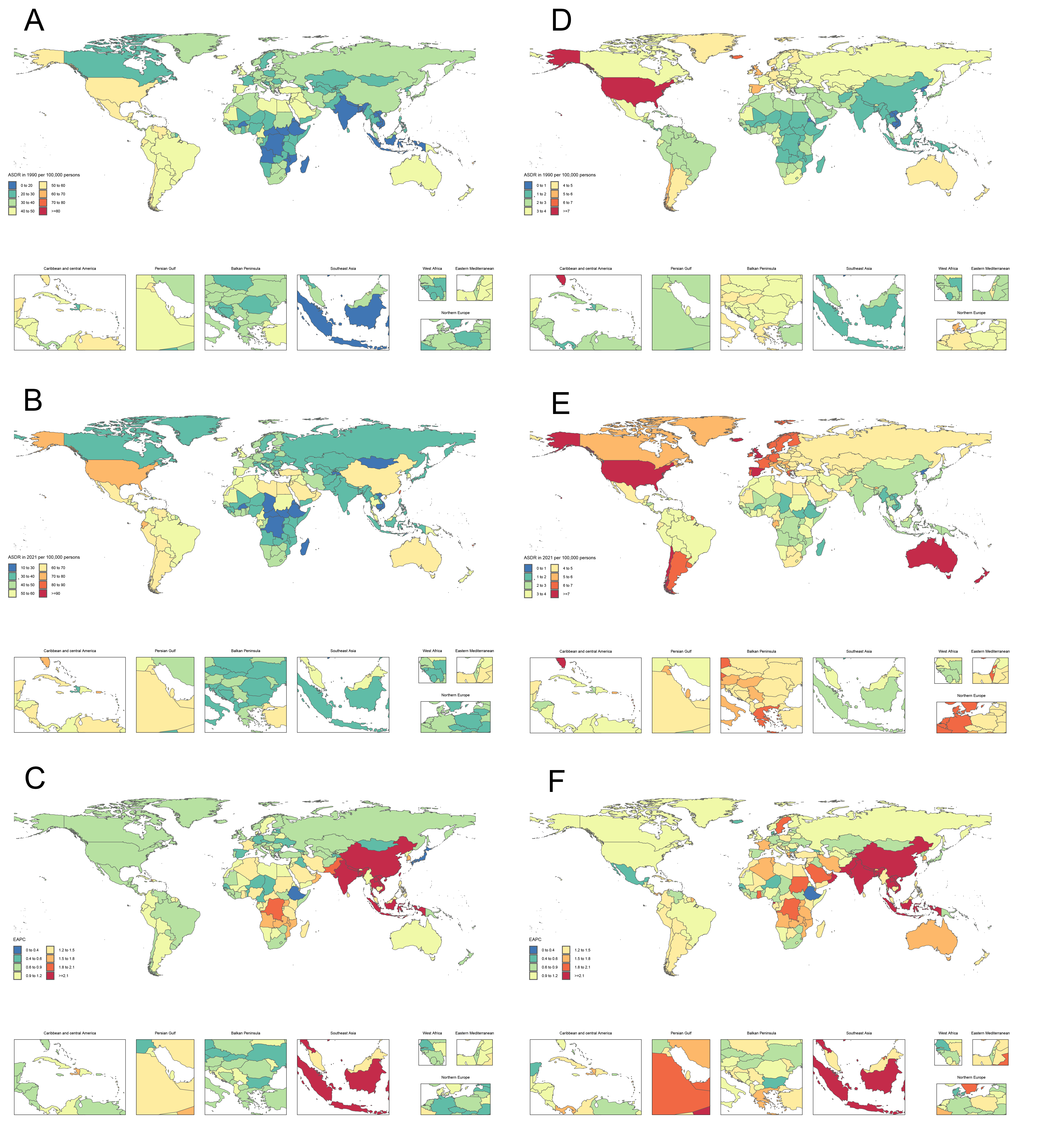
Fig S2.** The ASDR of early-onset osteoarthritis attributable to high BMI among female per 100,000 population in 1990 and 2021, by country, along with ASDR trends from 1990 to 2021 as measured by the EAPC.

**Note:** A, The ASDR of early-onset knee osteoarthritis attributable to high BMI among female in 1990, by country; B, The ASDR of early-onset knee osteoarthritis attributable to high among female BMI in 2021, by country; C, The trend in ASDR of early-onset knee osteoarthritis attributable to high BMI among female from 1990 to 2021; D, The ASDR of early-onset hip osteoarthritis attributable to high BMI among female in 1990, by country; E, The ASDR of early-onset hip osteoarthritis attributable to high BMI among female in 2021, by country; F, The trend in ASDR of early-onset hip osteoarthritis attributable to high BMI among female from 1990 to 2021.

**Abbreviations:** BMI, Body mass index; EAPC, estimated annual percentage change; ASDR, age-standardized disability-adjusted life years rate.
