# Supplementary material for: Global, regional, and national burden of early-onset OA attributable to high BMI: 1990–2021 estimates and 2036 projections from the global burden of disease study
Source: PLoS One. 2025 Jul 16;20(7):e0328414. doi: 10.1371/journal.pone.0328414 (PMC12266449; doi:10.1371/journal.pone.0328414)
Supplement: S4 Fig — Note: A, Early-onset knee osteoarthritis attributable to high BMI; B, Early-onset hip osteoarthritis attributable to high BMI. Abbreviations: BMI, Body mass index; ASDR, age-standardized disability-adjusted life years rate; GBD, Global Burden of Disease; SDI, Sociodemographic Index. (DOCX) [file pone.0328414.s005.docx]

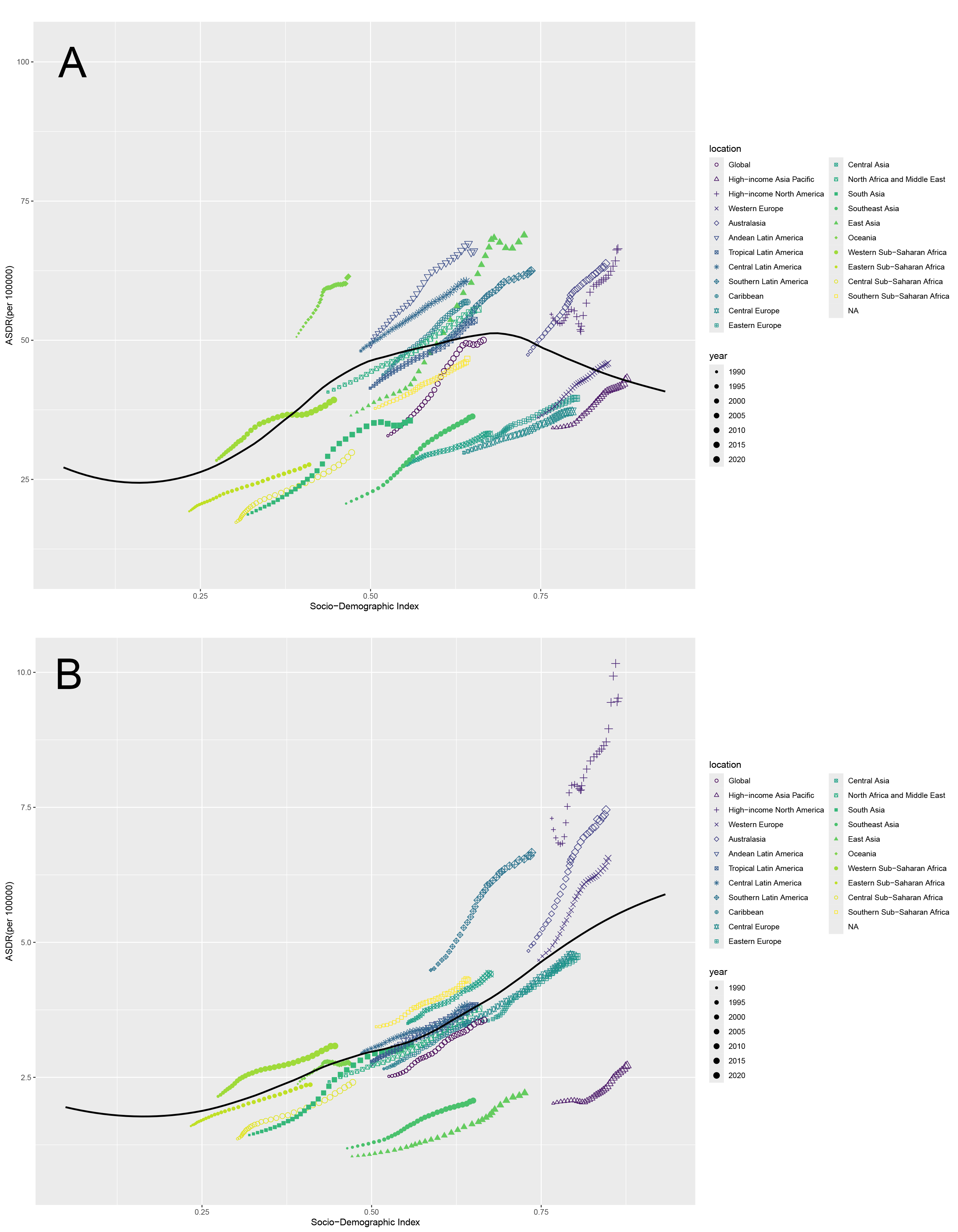


**Fig S4.** The association between the ASDR of female early-onset osteoarthritis attributable to high BMI among female and the SDI across the 21 GBD regions.

**Note:** A, Early-onset knee osteoarthritis attributable to high BMI; B, Early-onset hip osteoarthritis attributable to high BMI.

**Abbreviations:** BMI, Body mass index; ASDR, age-standardized disability-adjusted life years rate; GBD, Global Burden of Disease; SDI, Sociodemographic Index.
