# Supplementary material for: Global, regional, and national burden of early-onset OA attributable to high BMI: 1990–2021 estimates and 2036 projections from the global burden of disease study
Source: PLoS One. 2025 Jul 16;20(7):e0328414. doi: 10.1371/journal.pone.0328414 (PMC12266449; doi:10.1371/journal.pone.0328414)
Supplement: S6 Fig — Note: A, The association between the SDI and the ASDR of early-onset knee osteoarthritis attributable to high BMI among female across 204 countries in 1990; B, The association between the SDI and the ASDR of early-onset knee osteoarthritis attributable to high BMI among female across 204 countries in 2021; C, The association between the SDI and the ASDR of early-onset hip osteoarthritis attributable to high BMI among female across 204 countries in 1990; D, The association between the SDI and the ASDR of early-onset hip osteoarthritis attributable to high BMI among female across 204 countries in 2021. Abbreviations: BMI, Body mass index; ASDR, age-standardized disability-adjusted life years rate; SDI, Sociodemographic Index. (DOCX) [file pone.0328414.s007.docx]

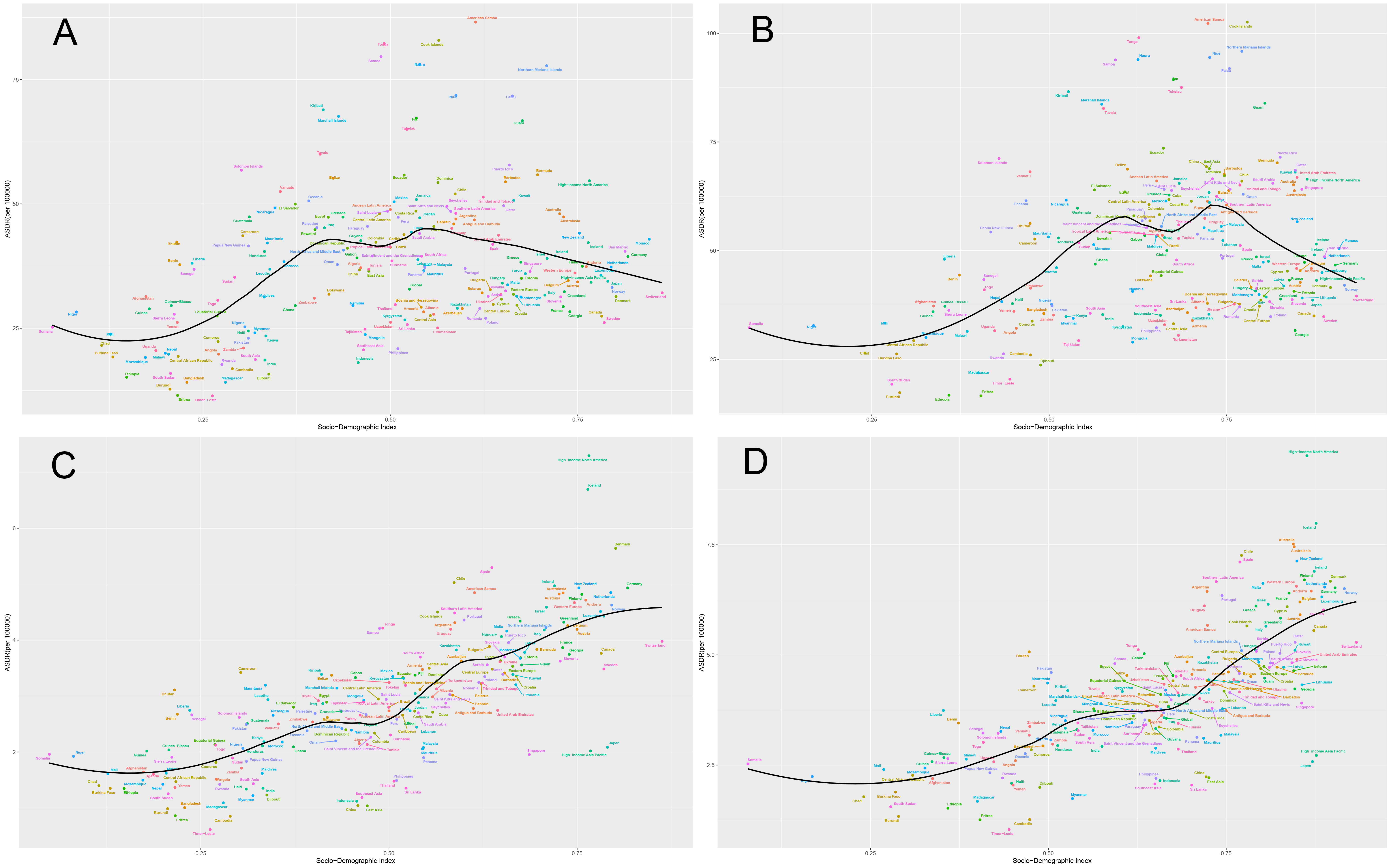


**Fig S6,** The association between the SDI and the ASDR of early-onset osteoarthritis attributable to high BMI among female across 204 countries.

**Note:** A, The association between the SDI and the ASDR of early-onset knee osteoarthritis attributable to high BMI among female across 204 countries in 1990; B, The association between the SDI and the ASDR of early-onset knee osteoarthritis attributable to high BMI among female across 204 countries in 2021; C, The association between the SDI and the ASDR of early-onset hip osteoarthritis attributable to high BMI among female across 204 countries in 1990; D, The association between the SDI and the ASDR of early-onset hip osteoarthritis attributable to high BMI among female across 204 countries in 2021.

**Abbreviations:** BMI, Body mass index; ASDR, age-standardized disability-adjusted life years rate; SDI, Sociodemographic Index.
