# Supplementary material for: Global, regional, and national burden of early-onset OA attributable to high BMI: 1990–2021 estimates and 2036 projections from the global burden of disease study
Source: PLoS One. 2025 Jul 16;20(7):e0328414. doi: 10.1371/journal.pone.0328414 (PMC12266449; doi:10.1371/journal.pone.0328414)
Supplement: S1 Table — (DOCX) [file pone.0328414.s008.docx]

| Table S1. The ASDR of early-onset osteoarthritis attributed to high BMI in 1990 and 2021 for male by SDI regions,and by GBD regions, with EAPC from 1990 to 2021. | | | | | | |
| --- | --- | --- | --- | --- | --- | --- |
|  | **Knee OA** | | | **Hip OA** | | |
| **Location** | **ASDR in 1990 (per 100,000)** | **ASDR in 2021 (per 100,000)** | **EAPC of ASDR (%) 1990–2021** | **ASDR in 1990 (per 100,000)** | **ASDR in 2021 (per 100,000)** | **EAPC of ASDR (%) 1990–2021** |
| Global | 20.91 (-1.9,58.2) | 32.04 (-3.13,86.53) | 1.54 (1.47,1.6) | 2.62 (-0.22,7.23) | 3.67 (-0.34,10.14) | 1.18 (1.13,1.22) |
| **SDI regions** | | | | | | |
| High SDI | 31.17 (-3.17,84.37) | 41.64 (-4.75,108.91) | 0.96 (0.92,1) | 5.53 (-0.52,15.23) | 7.44 (-0.76,20.04) | 1.17 (1.09,1.24) |
| High-middle SDI | 22.99 (-2.09,64.16) | 35.86 (-3.51,96.13) | 1.67 (1.57,1.77) | 3.01 (-0.25,8.31) | 4.15 (-0.38,11.43) | 1.08 (1.04,1.12) |
| Middle SDI | 19.73 (-1.68,55.75) | 33.17 (-3.14,90.46) | 1.91 (1.81,2.01) | 1.61 (-0.12,4.53) | 2.95 (-0.25,8.25) | 1.97 (1.96,1.99) |
| Low-middle SDI | 12.27 (-1.03,34.81) | 23.79 (-2.09,66.68) | 2.28 (2.21,2.34) | 1.07 (-0.08,3.02) | 2.26 (-0.18,6.37) | 2.49 (2.47,2.52) |
| Low SDI | 10.58 (-0.82,30.95) | 19.73 (-1.64,56.05) | 2.11 (2.08,2.14) | 0.99 (-0.07,2.89) | 1.94 (-0.14,5.53) | 2.23 (2.21,2.26) |
| **GBD regions** | | | | | | |
| Andean Latin America | 30.1 (-2.92,82.61) | 43.8 (-5.06,115.11) | 1.21 (1.19,1.24) | 2.86 (-0.23,8.05) | 4.37 (-0.44,11.88) | 1.43 (1.39,1.46) |
| Australasia | 34.2 (-3.59,92.84) | 48.78 (-5.59,129.64) | 1.15 (1.09,1.2) | 5.96 (-0.58,16.83) | 9.78 (-1.04,26.91) | 1.62 (1.52,1.72) |
| Caribbean | 28.14 (-2.55,76.59) | 39.61 (-4.1,105.69) | 1.15 (1.09,1.2) | 2.77 (-0.23,7.95) | 3.88 (-0.37,10.62) | 1.19 (1.13,1.25) |
| Central Asia | 20.82 (-2.06,57.14) | 25.33 (-2.73,67.53) | 0.66 (0.64,0.67) | 3.7 (-0.32,10.43) | 4.71 (-0.44,13) | 0.8 (0.78,0.83) |
| Central Europe | 26.61 (-2.62,72.17) | 31.55 (-3.35,83.77) | 0.56 (0.55,0.58) | 4.42 (-0.39,12.02) | 5.54 (-0.52,14.85) | 0.71 (0.69,0.74) |
| Central Latin America | 33.6 (-3.45,90.18) | 46.73 (-5.28,119.77) | 1.05 (1.03,1.07) | 3.44 (-0.32,9.56) | 4.79 (-0.49,12.94) | 0.98 (0.93,1.03) |
| Central Sub-Saharan Africa | 12.62 (-1.02,36.57) | 24.33 (-2.11,70.44) | 2.12 (2.08,2.17) | 1.34 (-0.1,4.29) | 2.67 (-0.19,8.04) | 2.2 (2.13,2.27) |
| East Asia | 19.58 (-1.63,56.64) | 36.96 (-3.31,102.07) | 2.53 (2.31,2.74) | 1.15 (-0.08,3.37) | 2.54 (-0.2,7.12) | 2.65 (2.6,2.7) |
| Eastern Europe | 24.62 (-2.18,68.27) | 33.04 (-3.32,87.39) | 1.01 (0.98,1.03) | 4.4 (-0.33,12.42) | 6.11 (-0.53,16.53) | 1.13 (1.1,1.15) |
| Eastern Sub-Saharan Africa | 11.01 (-0.86,31.79) | 19.32 (-1.58,55.22) | 1.83 (1.81,1.86) | 1.26 (-0.08,3.69) | 2.38 (-0.17,6.95) | 2.1 (2.05,2.15) |
| High-income Asia Pacific | 22.75 (-1.93,65.28) | 31.75 (-2.95,87.99) | 1.16 (1.09,1.23) | 2.89 (-0.23,8.22) | 3.99 (-0.32,11.21) | 1.09 (1.06,1.12) |
| High-income North America | 43.21 (-4.81,113.41) | 52.65 (-6.44,134.03) | 0.65 (0.51,0.78) | 8.52 (-0.85,23.43) | 11.44 (-1.23,30.9) | 1.43 (1.29,1.57) |
| North Africa and Middle East | 27.08 (-2.57,73.57) | 43.17 (-4.97,111.35) | 1.5 (1.48,1.52) | 2.36 (-0.22,6.72) | 4.16 (-0.43,11.3) | 1.8 (1.77,1.83) |
| Oceania | 30 (-2.73,82.14) | 37.95 (-4,101.19) | 0.71 (0.62,0.79) | 2.56 (-0.2,7.23) | 3.15 (-0.32,8.96) | 0.54 (0.4,0.67) |
| South Asia | 9.06 (-0.72,26.61) | 19.6 (-1.46,57.86) | 2.76 (2.65,2.87) | 0.67 (-0.05,1.97) | 1.68 (-0.12,4.79) | 3.16 (3.09,3.22) |
| Southeast Asia | 11.26 (-0.85,33.53) | 19.69 (-1.73,56.28) | 1.86 (1.79,1.94) | 1.11 (-0.07,3.26) | 2.04 (-0.14,5.9) | 2.03 (1.97,2.09) |
| Southern Latin America | 33.39 (-3.52,89.81) | 46.29 (-5.5,117.91) | 1.06 (0.98,1.13) | 5.34 (-0.52,14.96) | 8.64 (-0.99,24.14) | 1.55 (1.43,1.68) |
| Southern Sub-Saharan Africa | 25.93 (-2.17,72.9) | 38.96 (-3.56,105.2) | 1.29 (1.24,1.33) | 4.01 (-0.31,11.52) | 6.33 (-0.52,17.87) | 1.48 (1.45,1.51) |
| Tropical Latin America | 29.52 (-2.88,81.92) | 41.89 (-4.67,109.63) | 1.13 (1.1,1.16) | 3.18 (-0.27,8.67) | 4.74 (-0.47,13.08) | 1.34 (1.29,1.38) |
| Western Europe | 27.14 (-2.74,73.98) | 34.2 (-3.76,91.64) | 0.75 (0.71,0.8) | 5.51 (-0.5,15.04) | 7.4 (-0.73,20.05) | 0.96 (0.89,1.02) |
| Western Sub-Saharan Africa | 17.5 (-1.41,51.01) | 28.23 (-2.58,76.79) | 1.5 (1.42,1.59) | 1.85 (-0.13,5.33) | 2.99 (-0.23,8.29) | 1.38 (1.29,1.48) |
| **Country** | | | | | | |
| Afghanistan | 21.29 (-1.87,59.36) | 31.24 (-3.1,83.6) | 1.31 (1.26,1.37) | 1.58 (-0.11,4.74) | 2.5 (-0.23,7.52) | 1.63 (1.54,1.73) |
| Albania | 24.07 (-2.33,65.05) | 28.78 (-3.18,76.15) | 0.61 (0.59,0.63) | 3.59 (-0.3,10.23) | 4.55 (-0.44,12.6) | 0.83 (0.8,0.86) |
| Algeria | 21.54 (-2.08,61.33) | 39 (-4.09,103.19) | 1.97 (1.95,2) | 1.78 (-0.17,4.97) | 3.61 (-0.32,10.32) | 2.4 (2.38,2.43) |
| American Samoa | 59.33 (-7.22,147.89) | 67.81 (-8.27,165.46) | 0.36 (0.27,0.45) | 6.04 (-0.64,16.83) | 6.98 (-0.84,18.75) | 0.23 (0.05,0.41) |
| Andorra | 27.88 (-2.73,76.85) | 34.06 (-3.34,93.18) | 0.66 (0.64,0.67) | 5.5 (-0.49,16.01) | 7.28 (-0.64,20.07) | 0.89 (0.85,0.93) |
| Angola | 11.85 (-1,35.12) | 22.44 (-1.79,63.67) | 2.08 (2.06,2.1) | 1.22 (-0.08,3.76) | 2.56 (-0.18,7.73) | 2.36 (2.34,2.38) |
| Antigua and Barbuda | 31.53 (-3.34,86.16) | 44.33 (-5.18,116.45) | 1.11 (1.09,1.12) | 3.01 (-0.25,8.91) | 4.3 (-0.41,12.11) | 1.18 (1.16,1.2) |
| Argentina | 32.39 (-3.24,87.91) | 44.99 (-5.16,114.26) | 1.07 (0.99,1.14) | 5.12 (-0.5,14.96) | 8.32 (-0.93,23.01) | 1.57 (1.45,1.69) |
| Armenia | 19.87 (-2.03,55.56) | 24.86 (-2.61,68) | 0.77 (0.75,0.79) | 3.42 (-0.26,10.09) | 4.63 (-0.44,13.29) | 1.06 (1.03,1.08) |
| Australia | 34.26 (-3.58,93.68) | 49.65 (-5.57,131.65) | 1.19 (1.14,1.25) | 5.87 (-0.57,16.77) | 9.85 (-1.03,27.13) | 1.69 (1.59,1.8) |
| Austria | 26.48 (-2.49,72.25) | 32.9 (-3.2,90.52) | 0.68 (0.66,0.71) | 5.09 (-0.46,14.62) | 6.81 (-0.62,18.92) | 0.92 (0.89,0.96) |
| Azerbaijan | 20.3 (-2.23,55.36) | 25.42 (-2.76,66.31) | 0.78 (0.75,0.82) | 3.59 (-0.32,10.29) | 4.81 (-0.44,13.63) | 1.03 (0.97,1.1) |
| Bahamas | 36.58 (-3.91,99.21) | 49.02 (-5.93,128.56) | 0.96 (0.92,1.01) | 3.75 (-0.35,10.6) | 5.01 (-0.52,14.54) | 0.92 (0.87,0.96) |
| Bahrain | 34.73 (-3.67,96.25) | 50.2 (-6.47,127.39) | 1.2 (1.19,1.21) | 3.23 (-0.28,9.13) | 5.07 (-0.59,14.17) | 1.43 (1.4,1.46) |
| Bangladesh | 7.78 (-0.56,24.22) | 17.42 (-1.4,51.42) | 2.99 (2.85,3.13) | 0.48 (-0.03,1.54) | 1.27 (-0.08,3.96) | 3.64 (3.46,3.81) |
| Barbados | 35.02 (-3.44,97.55) | 47.98 (-5.09,123.34) | 1 (0.98,1.03) | 3.56 (-0.28,10.38) | 4.87 (-0.45,13.82) | 1.04 (1,1.08) |
| Belarus | 25.84 (-2.42,73.36) | 34.71 (-4.27,90.09) | 1.01 (0.99,1.03) | 3.87 (-0.31,11.31) | 5.41 (-0.61,14.96) | 1.16 (1.13,1.19) |
| Belgium | 24.38 (-2.23,69.24) | 32.37 (-3.19,88.61) | 0.89 (0.85,0.93) | 4.65 (-0.4,13.4) | 6.71 (-0.65,18.74) | 1.15 (1.09,1.2) |
| Belize | 37 (-3.85,99.63) | 49.89 (-5.41,127.71) | 0.94 (0.83,1.04) | 3.65 (-0.34,10.57) | 5.15 (-0.5,14.24) | 1.06 (0.9,1.23) |
| Benin | 17.99 (-1.34,53.11) | 31.76 (-2.87,89.15) | 1.79 (1.72,1.85) | 1.7 (-0.12,5.24) | 3.31 (-0.25,9.49) | 2.09 (1.99,2.2) |
| Bermuda | 41.82 (-4.89,108.55) | 54.4 (-6.8,141.4) | 0.85 (0.81,0.89) | 4.62 (-0.45,12.89) | 5.81 (-0.63,16.13) | 0.73 (0.69,0.77) |
| Bhutan | 22.73 (-2.14,63.38) | 36.3 (-3.28,98.36) | 1.54 (1.44,1.64) | 1.48 (-0.11,4.38) | 2.83 (-0.24,7.99) | 2.22 (2.09,2.35) |
| Bolivia (Plurinational State of) | 27.24 (-2.53,75.93) | 41.1 (-4.22,108.21) | 1.36 (1.32,1.39) | 2.48 (-0.19,7.32) | 3.97 (-0.4,11.06) | 1.6 (1.55,1.65) |
| Bosnia and Herzegovina | 24.13 (-2.6,68.35) | 29.91 (-3.49,78.96) | 0.71 (0.64,0.77) | 3.61 (-0.31,10.38) | 4.79 (-0.46,13.21) | 0.96 (0.84,1.07) |
| Botswana | 11.61 (-0.93,34.44) | 34.37 (-2.86,97.72) | 3.71 (3.61,3.81) | 1.47 (-0.09,4.61) | 4.88 (-0.37,14.37) | 4.09 (3.95,4.23) |
| Brazil | 29.47 (-2.88,81.73) | 41.86 (-4.67,109.29) | 1.13 (1.1,1.16) | 3.19 (-0.27,8.7) | 4.76 (-0.47,13.16) | 1.34 (1.3,1.39) |
| Brunei Darussalam | 29.81 (-2.52,87.81) | 44.24 (-4.32,122.17) | 1.33 (1.24,1.43) | 3.38 (-0.26,10.41) | 5.42 (-0.43,15.79) | 1.67 (1.56,1.79) |
| Bulgaria | 27.59 (-2.91,75.34) | 31.11 (-3.22,81.63) | 0.39 (0.37,0.41) | 4.52 (-0.39,12.86) | 5.22 (-0.45,14.67) | 0.42 (0.39,0.44) |
| Burkina Faso | 14.64 (-1.15,42.45) | 21.84 (-1.79,63.58) | 1.31 (1.29,1.32) | 1.36 (-0.1,4.19) | 2.13 (-0.16,6.64) | 1.43 (1.41,1.45) |
| Burundi | 7.74 (-0.65,23.86) | 11.74 (-0.95,34.4) | 1.35 (1.27,1.43) | 0.83 (-0.05,2.66) | 1.25 (-0.09,4.07) | 1.35 (1.25,1.45) |
| Cabo Verde | 18.22 (-1.48,53.51) | 33.49 (-3.21,91.88) | 2.02 (2.01,2.03) | 1.79 (-0.13,5.47) | 3.57 (-0.26,10.36) | 1.53 (1.5,1.57) |
| Cambodia | 8.36 (-0.71,24.63) | 14.46 (-1.13,43.22) | 1.88 (1.85,1.91) | 0.71 (-0.05,2.34) | 1.24 (-0.09,3.74) | 2.25 (2.23,2.28) |
| Cameroon | 27.63 (-2.45,78.22) | 41.67 (-4.13,108) | 1.31 (1.27,1.36) | 2.89 (-0.24,8.59) | 4.61 (-0.43,13.33) | 1.89 (1.84,1.95) |
| Canada | 21.24 (-2.19,56.93) | 28.22 (-3.32,75.42) | 1.05 (0.91,1.19) | 4.58 (-0.42,13.15) | 7.09 (-0.68,20.07) | 1.42 (1.38,1.46) |
| Central African Republic | 8.78 (-0.76,27.01) | 17.73 (-1.48,52.09) | 2.37 (2.33,2.4) | 0.92 (-0.06,2.82) | 1.88 (-0.14,5.67) | 1.39 (1.27,1.51) |
| Chad | 12.63 (-1,39.12) | 18.88 (-1.59,54.54) | 1.26 (1.24,1.27) | 1.13 (-0.08,3.8) | 1.73 (-0.14,5.33) | 2.45 (2.42,2.48) |
| Chile | 36.36 (-4.12,98.24) | 49.45 (-6.12,125.86) | 0.98 (0.91,1.04) | 5.95 (-0.58,16.47) | 9.43 (-1.12,26.27) | 1.34 (1.31,1.36) |
| China | 19.58 (-1.64,56.58) | 37.16 (-3.31,102.59) | 2.56 (2.34,2.78) | 1.15 (-0.08,3.38) | 2.56 (-0.21,7.17) | 1.45 (1.32,1.58) |
| Colombia | 28.33 (-2.54,77.45) | 42.4 (-4.54,112.81) | 1.27 (1.24,1.31) | 2.55 (-0.2,7.64) | 3.83 (-0.37,10.89) | 2.69 (2.63,2.74) |
| Comoros | 12.65 (-1.08,38.75) | 26.5 (-2.28,73.44) | 2.47 (2.45,2.49) | 1.45 (-0.11,4.51) | 3.11 (-0.26,9.26) | 1.31 (1.27,1.34) |
| Congo | 16.27 (-1.55,45.77) | 28.37 (-2.59,79.16) | 1.83 (1.8,1.85) | 1.85 (-0.15,5.62) | 3.44 (-0.28,9.98) | 2.6 (2.57,2.63) |
| Cook Islands | 55.86 (-6.78,140.57) | 67.29 (-9.42,167.45) | 0.56 (0.51,0.62) | 5.49 (-0.57,14.6) | 7.01 (-0.78,18.75) | 2.05 (2.02,2.08) |
| Costa Rica | 33.84 (-3.63,90.14) | 46.1 (-5.33,118.79) | 0.99 (0.96,1.01) | 3.08 (-0.28,9.36) | 4.25 (-0.39,12.02) | 0.64 (0.51,0.76) |
| Croatia | 28.34 (-3.02,76.19) | 34.33 (-3.87,88.69) | 0.66 (0.64,0.69) | 4.49 (-0.39,12.5) | 5.74 (-0.61,16.15) | 1.05 (1.01,1.08) |
| Cuba | 28.92 (-2.5,81.76) | 43.44 (-4.52,115.77) | 1.35 (1.32,1.39) | 2.79 (-0.22,8.16) | 4.29 (-0.43,12.07) | 0.84 (0.79,0.89) |
| Cyprus | 23.61 (-2.21,66.47) | 33.59 (-3.91,89.14) | 1.19 (1.12,1.26) | 4.37 (-0.38,12.74) | 6.79 (-0.61,19.2) | 1.5 (1.46,1.54) |
| Czechia | 30.15 (-3.18,79.68) | 34.27 (-3.66,89.35) | 0.42 (0.41,0.43) | 4.82 (-0.45,13.17) | 5.71 (-0.56,15.9) | 1.52 (1.44,1.61) |
| C么te d'Ivoire | 21.59 (-1.63,63.37) | 33.62 (-3.1,94.26) | 1.41 (1.39,1.43) | 2.07 (-0.17,6.48) | 3.37 (-0.26,9.83) | 0.53 (0.51,0.55) |
| Democratic People's Republic of Korea | 13.24 (-1.19,40.25) | 17.61 (-1.6,51.55) | 0.88 (0.79,0.97) | 0.74 (-0.05,2.24) | 0.93 (-0.06,2.83) | 0.67 (0.58,0.77) |
| Democratic Republic of the Congo | 12.6 (-1.02,36.25) | 24.53 (-2.1,71.27) | 2.14 (2.06,2.22) | 1.34 (-0.1,4.49) | 2.62 (-0.18,8.05) | 2.1 (1.99,2.22) |
| Denmark | 25.96 (-2.55,72.43) | 33.51 (-3.73,91.22) | 0.83 (0.8,0.85) | 6.86 (-0.59,20.29) | 7.38 (-0.76,21.44) | 0.19 (-0.02,0.41) |
| Djibouti | 8.55 (-0.67,26.08) | 16.01 (-1.15,47.6) | 2.07 (2.02,2.12) | 0.91 (-0.06,2.89) | 1.91 (-0.12,5.75) | 2.5 (2.43,2.58) |
| Dominica | 32.85 (-3.19,88.98) | 46.38 (-4.99,122.96) | 1.08 (1.03,1.12) | 3.15 (-0.27,9.19) | 4.51 (-0.46,12.4) | 1.14 (1.08,1.2) |
| Dominican Republic | 27.28 (-2.56,74.12) | 42.08 (-4.43,110.57) | 1.44 (1.38,1.5) | 2.59 (-0.21,8.12) | 4.14 (-0.43,11.83) | 1.65 (1.59,1.71) |
| Ecuador | 31.61 (-2.99,88.77) | 48.56 (-5.54,127.45) | 1.35 (1.31,1.39) | 3.18 (-0.23,8.94) | 5.04 (-0.57,14.16) | 1.51 (1.45,1.56) |
| Egypt | 31.29 (-2.9,81.71) | 47.78 (-5.5,124.87) | 1.31 (1.27,1.35) | 2.68 (-0.25,7.77) | 4.55 (-0.51,12.51) | 1.54 (1.46,1.62) |
| El Salvador | 35.17 (-3.94,93.18) | 47.37 (-5.41,121.1) | 0.92 (0.87,0.98) | 3.15 (-0.29,8.85) | 4.35 (-0.4,12.05) | 1 (0.92,1.09) |
| Equatorial Guinea | 16.57 (-1.31,48.44) | 35.29 (-3.36,95.9) | 2.62 (2.51,2.72) | 1.77 (-0.12,5.67) | 4.51 (-0.38,12.96) | 3.37 (3.22,3.53) |
| Eritrea | 4.9 (-0.4,15.82) | 12.09 (-0.87,36.72) | 3.06 (3.03,3.1) | 0.5 (-0.04,1.59) | 1.3 (-0.09,4.04) | 3.27 (3.22,3.33) |
| Estonia | 29.64 (-2.85,81.61) | 36.85 (-4.25,97.42) | 0.8 (0.74,0.86) | 4.6 (-0.41,13.06) | 5.93 (-0.62,16.81) | 0.98 (0.93,1.02) |
| Eswatini | 22.75 (-2.15,67) | 39.53 (-3.76,109.73) | 1.72 (1.55,1.89) | 3.15 (-0.24,9.43) | 5.91 (-0.52,16.95) | 1.88 (1.66,2.1) |
| Ethiopia | 9.83 (-0.72,27.85) | 16.88 (-1.41,48.41) | 1.81 (1.74,1.87) | 1.18 (-0.07,3.53) | 2.17 (-0.16,6.38) | 2.06 (2.03,2.1) |
| Fiji | 39.03 (-3.63,105.66) | 53.43 (-5.66,138.45) | 0.96 (0.9,1.02) | 3.49 (-0.28,10.01) | 4.99 (-0.48,14.16) | 1.07 (0.96,1.18) |
| Finland | 27.3 (-2.74,74.61) | 34.95 (-3.97,92.11) | 0.78 (0.74,0.82) | 5.34 (-0.47,15.05) | 7.28 (-0.72,19.95) | 1.01 (0.94,1.07) |
| France | 23.63 (-2.35,66.12) | 32.41 (-3.23,89.45) | 1.02 (0.98,1.06) | 4.78 (-0.41,13.73) | 7.05 (-0.62,20.42) | 1.29 (1.08,1.5) |
| Gabon | 21.76 (-1.76,62.58) | 38.07 (-3.71,102.98) | 1.8 (1.73,1.87) | 2.51 (-0.2,7.55) | 5.02 (-0.5,14.61) | 2.21 (2.11,2.31) |
| Gambia | 20.14 (-1.69,56.91) | 30.28 (-2.88,85.08) | 1.31 (1.25,1.37) | 1.94 (-0.15,5.73) | 3.08 (-0.23,9.16) | 1.47 (1.37,1.56) |
| Georgia | 23.72 (-2.28,64.86) | 26.5 (-2.79,69.18) | 0.41 (0.4,0.42) | 4.48 (-0.36,12.6) | 5.02 (-0.45,14.34) | 0.43 (0.41,0.45) |
| Germany | 29.49 (-3.19,80.38) | 35.06 (-4.01,92.98) | 0.55 (0.52,0.58) | 5.81 (-0.54,16.62) | 7.37 (-0.76,20.64) | 0.77 (0.73,0.81) |
| Ghana | 17.05 (-1.44,47.36) | 28.31 (-2.31,81.96) | 1.51 (1.46,1.57) | 1.63 (-0.12,4.91) | 3 (-0.23,9.02) | 1.85 (1.8,1.91) |
| Greece | 28.02 (-2.87,76.34) | 37.29 (-4.51,97.49) | 0.88 (0.79,0.96) | 4.97 (-0.49,14.35) | 6.98 (-0.74,19.61) | 1.85 (1.54,2.17) |
| Greenland | 23.75 (-2.53,64.82) | 29.76 (-3.15,79.28) | 0.79 (0.76,0.81) | 5 (-0.52,14.56) | 7.05 (-0.65,19.74) | 1.19 (1.13,1.24) |
| Grenada | 24.8 (-2.22,70.4) | 39.97 (-3.93,108.34) | 1.53 (1.48,1.59) | 2.32 (-0.17,6.9) | 3.83 (-0.35,10.97) | 1.63 (1.56,1.69) |
| Guam | 48.95 (-5.45,124.99) | 57.44 (-6.33,146.12) | 0.53 (0.51,0.55) | 4.69 (-0.51,13.33) | 5.66 (-0.58,15.74) | 0.6 (0.55,0.65) |
| Guatemala | 31.49 (-2.82,88.33) | 43.86 (-4.92,114.5) | 1.07 (1.06,1.07) | 2.66 (-0.23,7.62) | 3.79 (-0.37,11.02) | 1.16 (1.14,1.19) |
| Guinea | 13.86 (-1.14,40.1) | 22.36 (-1.84,61.73) | 1.52 (1.51,1.54) | 1.3 (-0.08,4.12) | 2.13 (-0.16,6.27) | 1.62 (1.59,1.64) |
| Guinea-Bissau | 14.61 (-1.15,41.59) | 24.76 (-2.05,71.51) | 1.67 (1.65,1.7) | 1.37 (-0.09,4.22) | 2.39 (-0.19,7.2) | 1.7 (1.66,1.75) |
| Guyana | 28.31 (-2.55,79.76) | 39.62 (-3.93,108.4) | 1.11 (1.08,1.14) | 2.72 (-0.22,8.19) | 3.86 (-0.37,11.19) | 1.15 (1.11,1.19) |
| Haiti | 11.39 (-0.93,33.18) | 20.8 (-1.77,60.19) | 2.06 (2.02,2.09) | 0.99 (-0.07,3.11) | 1.75 (-0.12,5.46) | 2.03 (1.98,2.08) |
| Honduras | 27.37 (-2.63,77.57) | 38.67 (-4.18,103.9) | 1.13 (1.07,1.18) | 2.28 (-0.17,6.94) | 3.35 (-0.34,9.26) | 1.26 (1.18,1.34) |
| Hungary | 30.46 (-3.21,80.71) | 35.44 (-3.84,92.68) | 0.51 (0.49,0.52) | 5.05 (-0.48,14.35) | 6.02 (-0.58,16.44) | 0.56 (0.52,0.6) |
| Iceland | 31.68 (-3.42,86.43) | 37.85 (-4.27,99.14) | 0.59 (0.57,0.61) | 7.52 (-0.75,21.7) | 8.83 (-0.95,25.05) | 0.42 (0.35,0.49) |
| India | 8.64 (-0.69,25.8) | 19.07 (-1.39,56.76) | 2.82 (2.7,2.95) | 0.63 (-0.04,1.87) | 1.61 (-0.11,4.6) | 3.17 (3.1,3.25) |
| Indonesia | 10.34 (-0.81,30.65) | 19.61 (-1.65,55.68) | 2.17 (2.04,2.29) | 1.08 (-0.07,3.23) | 2.18 (-0.15,6.46) | 2.37 (2.25,2.49) |
| Iran (Islamic Republic of) | 21.48 (-1.81,61.22) | 37.36 (-3.96,96.93) | 1.71 (1.65,1.77) | 2.23 (-0.17,6.36) | 4.11 (-0.4,11.37) | 1.83 (1.77,1.9) |
| Iraq | 36.21 (-3.71,96.38) | 43.42 (-4.79,112.12) | 0.6 (0.58,0.63) | 3.25 (-0.27,8.96) | 3.98 (-0.38,10.71) | 0.67 (0.62,0.71) |
| Ireland | 28.45 (-3.21,76.26) | 36.73 (-4.05,96.67) | 0.81 (0.76,0.87) | 5.52 (-0.59,15.46) | 7.8 (-0.81,21.44) | 1.07 (0.99,1.14) |
| Israel | 27.26 (-2.92,75.11) | 32.93 (-3.63,89.9) | 0.59 (0.55,0.62) | 4.98 (-0.46,14.11) | 6.46 (-0.61,18.3) | 0.77 (0.71,0.83) |
| Italy | 24.24 (-2.26,65.71) | 30.42 (-3.36,82.95) | 0.67 (0.62,0.72) | 4.95 (-0.42,13.81) | 6.57 (-0.61,18.01) | 0.92 (0.89,0.95) |
| Jamaica | 27.37 (-2.34,78.12) | 42.88 (-4.32,115) | 1.5 (1.41,1.6) | 2.6 (-0.24,7.65) | 4.19 (-0.33,12.19) | 1.61 (1.5,1.72) |
| Japan | 22.29 (-1.92,64.2) | 29.81 (-2.63,83.11) | 0.96 (0.93,0.99) | 2.98 (-0.23,8.63) | 4.01 (-0.32,11.57) | 1.01 (0.97,1.05) |
| Jordan | 34.55 (-4.02,92.35) | 48.7 (-5.89,123.79) | 1.1 (1.08,1.12) | 3 (-0.27,8.48) | 4.71 (-0.5,13.11) | 1.41 (1.35,1.47) |
| Kazakhstan | 21.31 (-2.11,58.86) | 26.6 (-2.77,71.79) | 0.69 (0.68,0.71) | 3.92 (-0.33,11.13) | 5.2 (-0.49,14.78) | 0.88 (0.85,0.91) |
| Kenya | 12.66 (-0.99,36.51) | 23.37 (-1.87,65.32) | 2 (1.92,2.09) | 1.69 (-0.12,5.04) | 3.44 (-0.25,9.87) | 2.35 (2.22,2.49) |
| Kiribati | 43.26 (-4.54,112.76) | 53.41 (-6.5,136.21) | 0.62 (0.53,0.7) | 3.81 (-0.4,10.56) | 4.75 (-0.44,12.83) | 0.54 (0.39,0.7) |
| Kuwait | 40.17 (-4.31,105.77) | 55 (-7.65,136.41) | 1.06 (1.04,1.08) | 3.83 (-0.38,10.85) | 5.76 (-0.66,15.63) | 1.35 (1.31,1.38) |
| Kyrgyzstan | 20.12 (-1.83,55.36) | 24.83 (-2.5,66.12) | 0.67 (0.65,0.69) | 3.48 (-0.27,10.35) | 4.44 (-0.41,12.45) | 0.72 (0.68,0.75) |
| Lao People's Democratic Republic | 8.89 (-0.69,27.12) | 18.03 (-1.35,52.99) | 2.56 (2.43,2.7) | 0.77 (-0.05,2.42) | 1.6 (-0.11,4.96) | 2.64 (2.52,2.76) |
| Latvia | 28.58 (-2.79,76.81) | 34.81 (-3.41,91.67) | 0.68 (0.66,0.69) | 4.44 (-0.39,13.12) | 5.65 (-0.52,16.35) | 0.8 (0.78,0.82) |
| Lebanon | 32.94 (-3.31,89.19) | 46.93 (-6.04,119.13) | 1.12 (1.08,1.15) | 2.74 (-0.22,7.62) | 4.29 (-0.43,11.89) | 1.36 (1.15,1.57) |
| Lesotho | 13.86 (-1.24,42.09) | 28.4 (-2.45,81.63) | 2.49 (2.44,2.54) | 1.72 (-0.12,5.25) | 3.85 (-0.24,11.5) | 2.8 (2.73,2.87) |
| Liberia | 28.61 (-2.61,79.84) | 40.29 (-4.13,107.05) | 1.19 (1.16,1.23) | 2.8 (-0.21,8.4) | 4.21 (-0.38,11.83) | 1.51 (1.44,1.57) |
| Libya | 30.55 (-3.01,84.97) | 46.43 (-5.73,117.73) | 1.36 (1.33,1.39) | 2.75 (-0.23,8.14) | 4.42 (-0.51,12.31) | 1.52 (1.47,1.57) |
| Lithuania | 27.64 (-2.87,76.47) | 34.89 (-4.27,91.16) | 0.79 (0.77,0.82) | 4.21 (-0.38,11.89) | 5.53 (-0.6,15.75) | 0.9 (0.84,0.96) |
| Luxembourg | 26.68 (-2.63,76.08) | 34.42 (-3.27,89.57) | 0.8 (0.77,0.83) | 5.29 (-0.47,15.57) | 7.29 (-0.64,20.67) | 1.02 (0.99,1.06) |
| Madagascar | 8.25 (-0.59,25.75) | 14.43 (-1.11,45.64) | 1.85 (1.79,1.91) | 0.88 (-0.05,2.85) | 1.56 (-0.1,5.21) | 1.94 (1.84,2.03) |
| Malawi | 12.5 (-1.21,37.36) | 20.64 (-1.73,57.37) | 1.57 (1.54,1.61) | 1.37 (-0.12,4.24) | 2.41 (-0.18,7.17) | 1.84 (1.8,1.88) |
| Malaysia | 20.05 (-1.79,57.47) | 27.55 (-2.66,75.63) | 0.98 (0.92,1.04) | 1.9 (-0.11,5.95) | 2.8 (-0.22,8.58) | 1.23 (1.16,1.29) |
| Maldives | 15.75 (-1.22,45.59) | 30.49 (-3.17,82.98) | 2.3 (2.22,2.38) | 1.34 (-0.09,4.2) | 2.91 (-0.25,8.58) | 2.64 (2.57,2.7) |
| Mali | 12.08 (-1.05,35.96) | 18.15 (-1.39,53.48) | 1.33 (1.31,1.35) | 1.11 (-0.08,3.38) | 1.73 (-0.13,5.25) | 1.44 (1.41,1.46) |
| Malta | 23.99 (-2.18,66.01) | 33.99 (-3.51,90.69) | 1.12 (1.03,1.2) | 4.57 (-0.38,13.19) | 7.16 (-0.74,20.68) | 1.42 (1.29,1.54) |
| Marshall Islands | 41.58 (-4.42,110.72) | 51.25 (-6.78,128.27) | 0.61 (0.56,0.66) | 3.49 (-0.31,9.83) | 4.43 (-0.45,11.98) | 0.62 (0.54,0.7) |
| Mauritania | 24.19 (-2,69.69) | 37.05 (-3.63,101.83) | 1.32 (1.26,1.37) | 2.47 (-0.17,7.39) | 4.06 (-0.36,11.68) | 1.51 (1.43,1.6) |
| Mauritius | 20.66 (-1.91,58.21) | 31.74 (-2.85,85.66) | 1.43 (1.36,1.51) | 1.95 (-0.15,5.72) | 3.15 (-0.27,9.38) | 1.57 (1.47,1.67) |
| Mexico | 36.19 (-3.94,97.87) | 49.18 (-5.7,125.02) | 0.96 (0.94,0.99) | 4.1 (-0.41,11.17) | 5.53 (-0.59,14.75) | 0.82 (0.74,0.9) |
| Micronesia (Federated States of) | 45.19 (-5.19,115.66) | 56.22 (-6.79,138.34) | 0.66 (0.59,0.73) | 4.1 (-0.37,12.09) | 5.11 (-0.55,13.79) | 0.58 (0.44,0.72) |
| Monaco | 32.37 (-3.26,86.7) | 38.47 (-4.22,102.83) | 0.54 (0.51,0.57) | 6.76 (-0.57,18.94) | 8.42 (-0.85,23.63) | 0.69 (0.65,0.73) |
| Mongolia | 19.17 (-1.82,53.07) | 22.87 (-2.28,63.55) | 0.46 (0.36,0.55) | 3.34 (-0.28,9.9) | 4.15 (-0.34,11.96) | 0.75 (0.7,0.79) |
| Montenegro | 31.72 (-3.47,81.01) | 35.82 (-4.11,91.6) | 0.47 (0.44,0.49) | 5.14 (-0.51,14.81) | 5.97 (-0.6,16.87) | 0.53 (0.5,0.56) |
| Morocco | 19.47 (-1.64,53.78) | 33.36 (-3.46,88.12) | 1.79 (1.77,1.81) | 1.56 (-0.12,4.64) | 2.97 (-0.25,8.43) | 2.13 (2.1,2.16) |
| Mozambique | 11.84 (-0.96,35.07) | 21.7 (-1.78,64.77) | 1.97 (1.93,2.01) | 1.25 (-0.08,3.75) | 2.45 (-0.19,7.46) | 2.22 (2.17,2.28) |
| Myanmar | 9.78 (-0.77,29.81) | 16.02 (-1.28,46.77) | 1.66 (1.57,1.75) | 0.85 (-0.06,2.7) | 1.4 (-0.1,4.36) | 1.77 (1.68,1.85) |
| Namibia | 17.33 (-1.31,49.52) | 32.69 (-2.61,92.91) | 2.1 (2.04,2.16) | 2.16 (-0.15,6.53) | 4.32 (-0.32,12.84) | 2.27 (2.2,2.33) |
| Nauru | 52.53 (-6.62,132.49) | 62.22 (-8.59,156.6) | 0.48 (0.45,0.52) | 4.87 (-0.51,13.62) | 5.82 (-0.74,15.7) | 0.4 (0.33,0.46) |
| Nepal | 12 (-0.83,36.56) | 26.25 (-2.43,72.47) | 2.68 (2.63,2.72) | 0.76 (-0.05,2.42) | 1.98 (-0.16,6.08) | 3.33 (3.25,3.42) |
| Netherlands | 27.05 (-2.69,74.28) | 34.71 (-3.83,91.71) | 0.8 (0.77,0.84) | 4.94 (-0.47,13.82) | 6.75 (-0.59,18.95) | 1.02 (0.98,1.07) |
| New Zealand | 33.89 (-3.52,89.89) | 44.28 (-5.57,114.85) | 0.9 (0.85,0.95) | 6.43 (-0.59,17.2) | 9.44 (-0.99,26.15) | 1.25 (1.18,1.33) |
| Nicaragua | 32.36 (-3.11,88.39) | 43.95 (-4.81,114.87) | 0.94 (0.9,0.99) | 2.82 (-0.22,8.34) | 3.95 (-0.4,11.43) | 1.01 (0.96,1.07) |
| Niger | 10.97 (-0.85,30.99) | 15.25 (-1.11,46.9) | 1.05 (1.04,1.06) | 1 (-0.08,3.05) | 1.41 (-0.09,4.47) | 1.09 (1.07,1.11) |
| Nigeria | 17.62 (-1.43,50.48) | 28.76 (-2.68,75.39) | 1.56 (1.39,1.72) | 2 (-0.14,5.64) | 3.17 (-0.25,8.77) | 1.23 (1.08,1.39) |
| Niue | 49.31 (-5.39,127.68) | 61.73 (-7.32,153.17) | 0.71 (0.65,0.77) | 4.65 (-0.47,12.71) | 6.09 (-0.69,16.44) | 0.78 (0.67,0.89) |
| North Macedonia | 28.57 (-2.73,76.5) | 32.89 (-3.8,86.5) | 0.5 (0.49,0.52) | 4.47 (-0.4,12.7) | 5.32 (-0.52,15.13) | 0.62 (0.59,0.64) |
| Northern Mariana Islands | 55.05 (-6.43,140.55) | 63.57 (-7.92,160.95) | 0.42 (0.35,0.49) | 5.37 (-0.6,14.98) | 6.18 (-0.72,16.37) | 0.27 (0.13,0.4) |
| Norway | 24.3 (-2.23,67.71) | 29.62 (-2.98,78.63) | 0.63 (0.61,0.64) | 5.27 (-0.44,14.93) | 6.84 (-0.62,18.67) | 0.86 (0.84,0.87) |
| Oman | 26.69 (-2.72,74.1) | 49.44 (-5.82,125.13) | 2.1 (2.05,2.15) | 2.16 (-0.2,6.38) | 4.8 (-0.49,13.48) | 2.74 (2.67,2.81) |
| Pakistan | 13.45 (-1.1,39.23) | 24.54 (-2.1,70.1) | 2.16 (2.08,2.24) | 1.22 (-0.09,3.66) | 2.52 (-0.2,7.25) | 2.61 (2.52,2.7) |
| Palau | 50.21 (-5.52,131.41) | 60.36 (-7.64,150.92) | 0.51 (0.44,0.58) | 4.86 (-0.47,13.55) | 6.02 (-0.68,16.95) | 0.51 (0.37,0.65) |
| Palestine | 32.3 (-3.15,88.87) | 43.61 (-4.58,110.26) | 0.95 (0.92,0.98) | 2.71 (-0.23,7.8) | 3.88 (-0.4,10.72) | 1.08 (1.02,1.14) |
| Panama | 21.53 (-1.92,61.57) | 40.06 (-3.71,109.48) | 2.09 (2.02,2.16) | 1.77 (-0.14,5.33) | 3.54 (-0.27,10.7) | 2.31 (2.25,2.38) |
| Papua New Guinea | 24.16 (-2.07,68.26) | 33.58 (-3.37,91.59) | 1.03 (0.95,1.1) | 1.95 (-0.14,5.97) | 2.65 (-0.25,7.95) | 0.89 (0.79,1) |
| Paraguay | 31.62 (-3.25,86.61) | 42.9 (-4.72,113.67) | 1.01 (0.99,1.04) | 2.92 (-0.24,8.34) | 3.99 (-0.38,11.03) | 1.06 (1.03,1.08) |
| Peru | 30.2 (-2.93,81.1) | 42.41 (-5.03,111.29) | 1.11 (1.09,1.13) | 2.81 (-0.24,8.14) | 4.17 (-0.41,11.46) | 1.35 (1.31,1.38) |
| Philippines | 12.48 (-0.92,35.71) | 19.54 (-1.46,55.94) | 1.44 (1.37,1.52) | 1.53 (-0.09,4.33) | 2.42 (-0.16,7.07) | 1.44 (1.39,1.5) |
| Poland | 24.41 (-2.56,64.77) | 28.83 (-3.33,76.89) | 0.54 (0.52,0.56) | 4.54 (-0.43,12.35) | 5.65 (-0.58,15.33) | 0.67 (0.64,0.71) |
| Portugal | 24.72 (-2.58,68.14) | 33.8 (-3.63,89.67) | 0.92 (0.83,1) | 4.66 (-0.43,13.89) | 6.95 (-0.69,19.58) | 1.08 (0.96,1.2) |
| Puerto Rico | 44.84 (-4.95,113.93) | 57.75 (-7.32,148.58) | 0.83 (0.78,0.89) | 4.9 (-0.47,14.02) | 6.46 (-0.67,17.95) | 0.98 (0.92,1.04) |
| Qatar | 41.35 (-5.05,108.67) | 55.66 (-7.43,142.23) | 0.93 (0.91,0.95) | 4.05 (-0.43,11.65) | 5.95 (-0.67,16.28) | 1.17 (1.1,1.24) |
| Republic of Korea | 24.2 (-2.1,68.21) | 34.66 (-3.51,96.76) | 1.27 (1.14,1.39) | 2.57 (-0.2,7.54) | 3.82 (-0.33,11.06) | 1.26 (1.17,1.34) |
| Republic of Moldova | 26.68 (-2.34,72.91) | 35.32 (-3.55,91.52) | 1 (0.97,1.03) | 3.89 (-0.32,11.45) | 5.62 (-0.53,15.9) | 1.3 (1.25,1.36) |
| Romania | 26.09 (-2.4,72.39) | 32.04 (-2.98,86.91) | 0.69 (0.67,0.72) | 4.03 (-0.32,11.62) | 5.34 (-0.4,14.9) | 0.95 (0.91,0.99) |
| Russian Federation | 23.95 (-2.13,66.3) | 32.99 (-3.21,87.05) | 1.09 (1.06,1.12) | 4.35 (-0.34,12.27) | 6.24 (-0.53,17.01) | 1.23 (1.19,1.26) |
| Rwanda | 9.8 (-0.8,29.22) | 15.91 (-1.25,47.01) | 1.57 (1.49,1.65) | 1.06 (-0.07,3.27) | 1.82 (-0.12,5.39) | 1.81 (1.7,1.92) |
| Saint Kitts and Nevis | 32.76 (-3.24,89.79) | 47.92 (-5.48,122.32) | 1.21 (1.17,1.26) | 3.3 (-0.27,9.59) | 4.87 (-0.49,13.93) | 1.3 (1.26,1.34) |
| Saint Lucia | 28.64 (-2.96,78.87) | 42.73 (-4.61,115.11) | 1.29 (1.24,1.33) | 2.72 (-0.21,7.86) | 4.2 (-0.38,11.88) | 1.38 (1.31,1.45) |
| Saint Vincent and the Grenadines | 23.75 (-2.5,67.64) | 37.29 (-3.66,100.81) | 1.5 (1.47,1.54) | 2.2 (-0.19,6.49) | 3.61 (-0.34,10.56) | 1.66 (1.62,1.7) |
| Samoa | 51.92 (-5.75,132.52) | 60.15 (-7.6,149.63) | 0.41 (0.35,0.46) | 4.88 (-0.52,13.49) | 5.7 (-0.7,15.27) | 0.27 (0.16,0.38) |
| San Marino | 30.34 (-2.8,82.29) | 37.22 (-4.36,99.59) | 0.64 (0.6,0.68) | 6.17 (-0.55,17.41) | 7.95 (-0.78,21.39) | 0.81 (0.77,0.86) |
| Sao Tome and Principe | 18.87 (-1.72,54.78) | 32.76 (-2.94,91.53) | 1.81 (1.8,1.82) | 1.85 (-0.15,5.69) | 3.41 (-0.28,10.11) | 2 (1.98,2.03) |
| Saudi Arabia | 33.03 (-3.42,88.46) | 51.65 (-6.67,128.78) | 1.42 (1.37,1.47) | 2.87 (-0.25,8.62) | 5.26 (-0.59,14.28) | 1.92 (1.86,1.99) |
| Senegal | 17.16 (-1.44,51.3) | 25.78 (-2.31,70.58) | 1.23 (1.19,1.26) | 1.65 (-0.12,5.12) | 2.58 (-0.19,8) | 1.33 (1.28,1.38) |
| Serbia | 25.09 (-2.43,69.46) | 31.95 (-3.55,83.8) | 0.82 (0.8,0.84) | 3.96 (-0.36,11.19) | 5.36 (-0.53,15.04) | 1.02 (1,1.05) |
| Seychelles | 23.14 (-2.16,64.66) | 37.66 (-4.48,100.82) | 1.58 (1.48,1.67) | 2.27 (-0.18,6.74) | 3.8 (-0.4,11.1) | 1.63 (1.53,1.73) |
| Sierra Leone | 13.56 (-1.12,40.22) | 23.19 (-1.88,65.7) | 1.71 (1.59,1.82) | 1.26 (-0.09,3.97) | 2.25 (-0.16,7.32) | 1.82 (1.69,1.95) |
| Singapore | 26.22 (-2.21,75.4) | 40.86 (-3.62,113.76) | 1.35 (1.24,1.46) | 2.93 (-0.2,8.56) | 5 (-0.4,14.15) | 1.7 (1.58,1.82) |
| Slovakia | 29.45 (-3.17,76.05) | 33.65 (-3.53,88.6) | 0.4 (0.38,0.42) | 4.72 (-0.46,13.11) | 5.62 (-0.55,15.79) | 0.48 (0.43,0.52) |
| Slovenia | 28.47 (-2.97,75.41) | 33.49 (-3.83,87.2) | 0.57 (0.56,0.58) | 4.56 (-0.38,12.67) | 5.7 (-0.57,15.52) | 0.73 (0.7,0.76) |
| Solomon Islands | 30.62 (-2.64,85.17) | 41.77 (-4.73,112.53) | 0.95 (0.89,1.01) | 2.53 (-0.18,7.5) | 3.52 (-0.35,10.03) | 0.92 (0.79,1.04) |
| Somalia | 9.77 (-0.81,29.08) | 15.33 (-1.26,44.46) | 1.5 (1.43,1.57) | 1.04 (-0.07,3.22) | 1.65 (-0.12,5.03) | 1.56 (1.48,1.64) |
| South Africa | 29.78 (-2.53,83.23) | 42.5 (-4.01,112.74) | 1.11 (1.08,1.14) | 4.74 (-0.37,13.63) | 7.15 (-0.61,20.15) | 1.34 (1.3,1.37) |
| South Sudan | 8.46 (-0.71,25.56) | 13.55 (-1.08,40.31) | 1.52 (1.43,1.61) | 0.93 (-0.06,2.93) | 1.54 (-0.11,4.81) | 1.64 (1.53,1.75) |
| Spain | 26.38 (-2.81,72.39) | 34.3 (-3.9,92.53) | 1.02 (0.93,1.11) | 5.71 (-0.52,15.9) | 8.04 (-0.87,22.48) | 0.96 (0.81,1.11) |
| Sri Lanka | 13.85 (-1.08,42.63) | 21.94 (-1.75,64.68) | 1.52 (1.5,1.55) | 1.23 (-0.08,3.9) | 2.04 (-0.12,6.4) | 1.67 (1.63,1.71) |
| Sudan | 21.17 (-1.79,60.51) | 37.52 (-3.85,99.05) | 1.87 (1.85,1.89) | 1.58 (-0.11,4.68) | 3.26 (-0.3,9.52) | 2.39 (2.35,2.44) |
| Suriname | 26.46 (-2.34,74.62) | 38.6 (-4.07,105.35) | 1.23 (1.22,1.25) | 2.6 (-0.2,7.81) | 3.82 (-0.35,10.94) | 1.33 (1.31,1.36) |
| Sweden | 20.85 (-2.18,55.47) | 26.6 (-3.05,74.1) | 0.81 (0.78,0.84) | 5.45 (-0.49,15.17) | 7.83 (-0.73,22.47) | 1.04 (0.98,1.09) |
| Switzerland | 25.67 (-2.51,70.95) | 29.98 (-3.19,82.91) | 0.53 (0.5,0.57) | 4.93 (-0.4,14.25) | 6.05 (-0.56,17.58) | 0.68 (0.64,0.72) |
| Syrian Arab Republic | 30.51 (-3.29,82.75) | 46.54 (-6.27,117.93) | 1.37 (1.33,1.41) | 2.54 (-0.23,7.44) | 4.2 (-0.45,11.65) | 1.53 (1.45,1.6) |
| Taiwan (Province of China) | 28.12 (-2.36,81.01) | 46.57 (-5.11,126.1) | 1.77 (1.72,1.81) | 1.66 (-0.12,4.85) | 2.81 (-0.24,8.29) | 1.85 (1.78,1.92) |
| Tajikistan | 19.76 (-1.87,53.89) | 23.36 (-2.7,61.89) | 0.54 (0.52,0.55) | 3.27 (-0.25,9.44) | 3.91 (-0.34,10.95) | 0.57 (0.53,0.6) |
| Thailand | 14.75 (-1.13,43.91) | 28.09 (-2.86,80.33) | 2.17 (2.07,2.26) | 1.25 (-0.08,3.93) | 2.56 (-0.22,7.81) | 2.43 (2.33,2.52) |
| Timor-Leste | 5.64 (-0.49,17.63) | 11.51 (-0.81,34.59) | 2.55 (2.48,2.62) | 0.48 (-0.04,1.53) | 0.95 (-0.06,2.89) | 2.41 (2.33,2.5) |
| Togo | 13.41 (-1.05,39.42) | 24.06 (-1.75,69.68) | 1.83 (1.79,1.86) | 1.27 (-0.09,3.93) | 2.36 (-0.17,7.25) | 1.91 (1.85,1.97) |
| Tokelau | 46.94 (-5.2,122.61) | 60.09 (-7.63,150.02) | 0.79 (0.74,0.84) | 4.18 (-0.4,11.67) | 5.73 (-0.69,15.7) | 0.92 (0.82,1.02) |
| Tonga | 51.33 (-5.8,135.02) | 62.84 (-8.72,157.49) | 0.59 (0.5,0.67) | 4.75 (-0.47,13.17) | 5.98 (-0.69,15.84) | 0.52 (0.34,0.69) |
| Trinidad and Tobago | 33.54 (-3.17,89.31) | 46.19 (-5.33,123.26) | 1.06 (1.04,1.08) | 3.38 (-0.28,9.68) | 4.65 (-0.5,13.37) | 1.14 (1.11,1.18) |
| Tunisia | 25.45 (-2.4,70.69) | 40.06 (-4.53,104.72) | 1.47 (1.45,1.5) | 2.12 (-0.17,6.13) | 3.62 (-0.34,10.03) | 1.74 (1.72,1.77) |
| Turkmenistan | 21.69 (-2.28,58.96) | 25.14 (-2.88,66.19) | 0.5 (0.48,0.52) | 3.84 (-0.36,11.03) | 4.81 (-0.43,14.1) | 1.61 (1.57,1.64) |
| Tuvalu | 45.51 (-5.83,117.07) | 58.15 (-7.61,147.3) | 0.74 (0.69,0.79) | 4.01 (-0.43,11.03) | 5.29 (-0.63,14.66) | 0.72 (0.69,0.75) |
| Turkey | 30.33 (-3.01,82.5) | 46.01 (-5.84,120.67) | 1.36 (1.32,1.39) | 2.61 (-0.22,7.89) | 4.29 (-0.47,11.44) | 0.77 (0.67,0.86) |
| Uganda | 11.7 (-0.96,35.63) | 19.86 (-1.68,56.78) | 1.72 (1.71,1.74) | 1.25 (-0.09,4.01) | 2.26 (-0.16,7.07) | 1.97 (1.94,2.01) |
| Ukraine | 25.46 (-2.17,70.22) | 32.37 (-3.34,87.41) | 0.83 (0.81,0.84) | 4.67 (-0.33,13.6) | 5.93 (-0.54,16.61) | 0.85 (0.83,0.87) |
| United Arab Emirates | 34.43 (-3.11,93.26) | 54.29 (-6.65,136) | 1.5 (1.47,1.53) | 3.12 (-0.3,8.88) | 5.44 (-0.68,14.62) | 1.91 (1.87,1.95) |
| United Kingdom | 32.44 (-3.47,87.41) | 40.52 (-4.68,104.48) | 0.74 (0.68,0.8) | 6.84 (-0.64,18.58) | 8.92 (-0.92,24.01) | 0.88 (0.84,0.93) |
| United Republic of Tanzania | 13.68 (-1.13,41.08) | 23.65 (-1.96,66.72) | 1.73 (1.7,1.75) | 1.49 (-0.1,4.61) | 2.84 (-0.19,8.59) | 2.01 (1.98,2.05) |
| United States of America | 45.76 (-5.13,120.27) | 55.47 (-6.8,141.08) | 0.62 (0.47,0.77) | 8.98 (-0.91,24.6) | 11.94 (-1.29,31.99) | 1.43 (1.28,1.59) |
| United States Virgin Islands | 42.53 (-4.58,110.62) | 52.18 (-5.53,136.61) | 0.66 (0.62,0.69) | 4.69 (-0.46,13.49) | 5.75 (-0.55,16.1) | 0.67 (0.63,0.72) |
| Uruguay | 32.78 (-3.16,89.02) | 45 (-5.23,115.98) | 1.01 (0.94,1.08) | 5.32 (-0.49,15.17) | 8.37 (-0.83,23.43) | 1.53 (1.43,1.64) |
| Uzbekistan | 19.86 (-1.99,54.64) | 25.33 (-2.76,66.47) | 0.86 (0.84,0.88) | 3.4 (-0.29,10.34) | 4.67 (-0.43,13.25) | 1.08 (1.04,1.11) |
| Vanuatu | 31.73 (-3.18,87.42) | 42.35 (-4.55,113.3) | 0.95 (0.9,1) | 2.69 (-0.22,7.89) | 3.59 (-0.34,10.56) | 0.92 (0.86,0.99) |
| Venezuela (Bolivarian Republic of) | 35.53 (-3.7,94.71) | 47.25 (-5,120.62) | 0.94 (0.93,0.95) | 3.26 (-0.27,9.82) | 4.32 (-0.4,12.27) | 0.94 (0.91,0.98) |
| Viet Nam | 5.94 (-0.43,18.18) | 12.73 (-0.98,38.41) | 2.82 (2.65,3) | 0.5 (-0.03,1.55) | 1.09 (-0.08,3.43) | 2.95 (2.75,3.15) |
| Yemen | 14.75 (-1.02,44.06) | 28.54 (-2.59,78.1) | 2.26 (2.22,2.31) | 1.07 (-0.07,3.57) | 2.2 (-0.17,6.08) | 2.5 (2.45,2.56) |
| Zambia | 15.03 (-1.43,43.05) | 26.15 (-2.38,74.1) | 1.76 (1.7,1.82) | 1.7 (-0.12,5.1) | 3.08 (-0.21,9.46) | 1.93 (1.83,2.02) |
| Zimbabwe | 13.77 (-0.98,41.04) | 23.23 (-1.76,66.18) | 1.5 (1.4,1.59) | 1.69 (-0.11,5.17) | 2.94 (-0.21,8.67) | 1.53 (1.42,1.64) |
