# Supplementary material for: Global, regional, and national burden of early-onset OA attributable to high BMI: 1990–2021 estimates and 2036 projections from the global burden of disease study
Source: PLoS One. 2025 Jul 16;20(7):e0328414. doi: 10.1371/journal.pone.0328414 (PMC12266449; doi:10.1371/journal.pone.0328414)
Supplement: S2 Table — (DOCX) [file pone.0328414.s009.docx]

| Table S2. The ASDR of early-onset osteoarthritis attributed to high BMI in 1990 and 2021 for female by SDI quintiles and by GBD regions, with EAPC from 1990 to 2021. | | | | | | |
| --- | --- | --- | --- | --- | --- | --- |
|  | **Knee OA** | | | **Hip OA** | | |
| **Location** | **ASDR in 1990 (per 100,000)** | **ASDR in 2021 (per 100,000)** | **EAPC of ASDR (%) 1990–2021** | **ASDR in 1990 (per 100,000)** | **ASDR in 2021 (per 100,000)** | **EAPC of ASDR (%) 1990–2021** |
| Global | 32.85 (-2.9,90.5) | 50.02 (-4.97,134.26) | 1.56 (1.48,1.63) | 2.52 (-0.2,6.92) | 3.56 (-0.32,9.77) | 1.22 (1.19,1.25) |
| **SDI regions** | | | | | | |
| High SDI | 41.46 (-3.85,114.41) | 55.23 (-5.78,145.06) | 0.99 (0.95,1.04) | 4.61 (-0.39,12.76) | 6.32 (-0.6,17.37) | 1.17 (1.1,1.23) |
| High-middle SDI | 34.93 (-3.2,96.33) | 57.02 (-5.75,152.08) | 1.81 (1.72,1.91) | 2.69 (-0.22,7.48) | 3.62 (-0.34,9.92) | 1.01 (0.95,1.08) |
| Middle SDI | 33.95 (-2.92,94.46) | 55.31 (-5.42,148.72) | 1.82 (1.72,1.92) | 1.65 (-0.13,4.59) | 2.94 (-0.26,8.09) | 1.97 (1.93,2.02) |
| Low-middle SDI | 22.95 (-2,62.9) | 38.76 (-3.82,104.58) | 1.89 (1.81,1.98) | 1.59 (-0.12,4.5) | 3.03 (-0.27,8.33) | 2.35 (2.27,2.44) |
| Low SDI | 19.37 (-1.5,54.25) | 28.71 (-2.5,79.64) | 1.36 (1.3,1.42) | 1.44 (-0.1,4.08) | 2.24 (-0.17,6.33) | 1.57 (1.49,1.65) |
| **GBD regions** | | | | | | |
| Andean Latin America | 48.88 (-5.07,129.6) | 66.03 (-7.6,169.58) | 1.09 (1.04,1.14) | 2.73 (-0.24,7.48) | 3.84 (-0.37,10.57) | 1.14 (1.12,1.17) |
| Australasia | 47.39 (-4.59,129.63) | 63.78 (-6.98,166.61) | 0.96 (0.91,1.01) | 4.84 (-0.41,14.16) | 7.45 (-0.69,21.29) | 1.46 (1.37,1.55) |
| Caribbean | 43.83 (-4.12,117.3) | 56.86 (-6.35,148.24) | 0.9 (0.87,0.94) | 2.66 (-0.22,7.45) | 3.51 (-0.33,9.56) | 1.01 (0.96,1.05) |
| Central Asia | 27.54 (-2.67,74.12) | 33.18 (-3.68,85.42) | 0.62 (0.61,0.63) | 3.5 (-0.32,9.59) | 4.41 (-0.43,12.1) | 0.81 (0.79,0.83) |
| Central Europe | 29.8 (-2.79,81.09) | 37.22 (-3.66,97.94) | 0.75 (0.73,0.77) | 3.48 (-0.28,9.66) | 4.75 (-0.42,12.97) | 1.06 (1.03,1.09) |
| Central Latin America | 48.08 (-4.86,127.56) | 60.6 (-7.01,154.21) | 0.75 (0.74,0.76) | 2.95 (-0.27,8.18) | 3.84 (-0.39,10.4) | 0.79 (0.74,0.83) |
| Central Sub-Saharan Africa | 17.3 (-1.32,50.76) | 29.83 (-2.5,83.04) | 1.74 (1.68,1.8) | 1.36 (-0.09,4.12) | 2.41 (-0.18,6.98) | 1.81 (1.72,1.89) |
| East Asia | 36.47 (-3.12,105.63) | 68.88 (-6.48,187.27) | 2.46 (2.28,2.64) | 1.04 (-0.08,3.06) | 2.21 (-0.18,6.27) | 2.61 (2.51,2.71) |
| Eastern Europe | 31.82 (-3.15,84.6) | 39.58 (-4.68,101.31) | 0.75 (0.73,0.77) | 3.53 (-0.29,9.7) | 4.74 (-0.48,13.28) | 1.04 (1.02,1.07) |
| Eastern Sub-Saharan Africa | 19.27 (-1.41,54.03) | 27.66 (-2.34,75.92) | 1.19 (1.17,1.21) | 1.6 (-0.1,4.67) | 2.36 (-0.18,6.71) | 1.32 (1.29,1.35) |
| High-income Asia Pacific | 34.25 (-3,98.8) | 43.16 (-4,123.09) | 0.82 (0.77,0.86) | 2.02 (-0.15,5.73) | 2.72 (-0.21,7.89) | 1.01 (0.89,1.13) |
| High-income North America | 54.66 (-5.34,146.59) | 66.44 (-7.7,169.91) | 0.7 (0.55,0.85) | 7.3 (-0.63,20.13) | 9.52 (-0.96,25.94) | 1.14 (1.02,1.27) |
| North Africa and Middle East | 40.71 (-4.29,108.53) | 55.57 (-6.8,139.98) | 1.02 (1,1.03) | 2.42 (-0.23,6.65) | 3.77 (-0.41,9.9) | 1.42 (1.4,1.45) |
| Oceania | 50.61 (-4.94,134.38) | 61.47 (-6.68,167.94) | 0.56 (0.47,0.64) | 2.38 (-0.2,6.71) | 2.76 (-0.24,7.79) | 0.39 (0.28,0.5) |
| South Asia | 18.71 (-1.43,53.4) | 35.58 (-3.14,100.52) | 2.43 (2.28,2.58) | 1.43 (-0.1,4.2) | 3.11 (-0.25,8.71) | 2.96 (2.79,3.13) |
| Southeast Asia | 20.67 (-1.64,59.53) | 36.31 (-3.17,100.12) | 1.92 (1.85,2) | 1.19 (-0.08,3.44) | 2.07 (-0.16,5.71) | 1.92 (1.85,1.99) |
| Southern Latin America | 48.11 (-4.87,129.39) | 62.48 (-6.97,157.32) | 0.88 (0.83,0.93) | 4.49 (-0.42,12.5) | 6.66 (-0.65,18.21) | 1.35 (1.25,1.45) |
| Southern Sub-Saharan Africa | 37.76 (-3.84,99.58) | 46.67 (-5.6,116.51) | 0.67 (0.67,0.68) | 3.44 (-0.29,9.41) | 4.3 (-0.45,11.57) | 0.75 (0.72,0.78) |
| Tropical Latin America | 41.39 (-3.95,110.82) | 53.59 (-5.75,141) | 0.84 (0.82,0.85) | 2.8 (-0.24,7.88) | 3.82 (-0.34,10.61) | 1.06 (1.04,1.08) |
| Western Europe | 36.13 (-3.24,99.91) | 45.89 (-4.4,122.71) | 0.77 (0.72,0.81) | 4.67 (-0.38,12.95) | 6.56 (-0.6,18.1) | 1.09 (1.01,1.18) |
| Western Sub-Saharan Africa | 28.43 (-2.48,78) | 39.28 (-3.82,105.44) | 1.03 (0.96,1.1) | 2.15 (-0.16,6.07) | 3.08 (-0.26,8.66) | 1.11 (1.06,1.16) |
| **Country** | | | | | | |
| Afghanistan | 31.37 (-2.63,85.54) | 37.26 (-3.37,100.93) | 0.65 (0.58,0.71) | 1.64 (-0.12,5.01) | 2.19 (-0.16,6.12) | 1.07 (0.96,1.18) |
| Albania | 27.82 (-2.82,77.08) | 36.86 (-4.3,95.91) | 0.95 (0.93,0.97) | 2.99 (-0.28,8.83) | 4.26 (-0.41,11.89) | 1.25 (1.22,1.28) |
| Algeria | 37.17 (-3.21,104.13) | 53.53 (-6.16,136.49) | 1.22 (1.17,1.28) | 2.15 (-0.16,6.32) | 3.63 (-0.37,9.84) | 1.73 (1.67,1.79) |
| American Samoa | 86.58 (-11.89,220.58) | 102.3 (-15.19,254.32) | 0.45 (0.38,0.53) | 4.85 (-0.55,13.19) | 5.67 (-0.74,15.2) | 0.34 (0.18,0.5) |
| Andorra | 37.4 (-3.77,105.59) | 45.89 (-4.66,123.93) | 0.69 (0.66,0.71) | 4.71 (-0.41,13.9) | 6.45 (-0.56,18.29) | 1.03 (0.97,1.08) |
| Angola | 19.78 (-1.44,56.18) | 32.13 (-3,90.15) | 1.57 (1.51,1.63) | 1.51 (-0.11,4.67) | 2.6 (-0.2,7.84) | 1.78 (1.69,1.87) |
| Antigua and Barbuda | 46.79 (-4.98,125.57) | 60.49 (-6.97,156.48) | 0.85 (0.83,0.87) | 2.78 (-0.29,7.76) | 3.74 (-0.34,10.84) | 0.98 (0.96,1) |
| Argentina | 46.97 (-4.84,126.06) | 61.34 (-6.6,153.67) | 0.91 (0.86,0.96) | 4.31 (-0.42,12.08) | 6.44 (-0.6,17.58) | 1.38 (1.28,1.48) |
| Armenia | 28.3 (-2.78,74.4) | 33.93 (-3.96,86.03) | 0.61 (0.59,0.63) | 3.47 (-0.32,9.91) | 4.56 (-0.48,13.01) | 0.95 (0.91,0.99) |
| Australia | 48.03 (-4.72,130.05) | 65.15 (-7.24,170.45) | 0.98 (0.92,1.04) | 4.82 (-0.4,14.01) | 7.52 (-0.7,21.56) | 1.5 (1.4,1.6) |
| Austria | 34.32 (-3.43,96.61) | 42.7 (-3.7,120.6) | 0.69 (0.67,0.71) | 4.19 (-0.36,12.44) | 5.91 (-0.46,17.49) | 1.03 (1,1.05) |
| Azerbaijan | 28.97 (-3.04,78.52) | 35.74 (-3.89,89.14) | 0.76 (0.73,0.78) | 3.63 (-0.32,10.22) | 4.84 (-0.46,13.35) | 1.08 (1.02,1.13) |
| Bahamas | 52.71 (-4.72,143.02) | 65.64 (-7.35,169.11) | 0.72 (0.69,0.75) | 3.38 (-0.28,9.5) | 4.18 (-0.38,11.85) | 0.72 (0.69,0.76) |
| Bahrain | 45.98 (-4.48,121.52) | 63.84 (-8.97,160.61) | 1.06 (1.05,1.08) | 3.02 (-0.23,8.58) | 4.61 (-0.59,12.43) | 1.43 (1.38,1.47) |
| Bangladesh | 14.13 (-1.08,43.63) | 35.54 (-3.08,99.65) | 3.5 (3.35,3.66) | 1 (-0.07,3.3) | 2.93 (-0.24,8.54) | 4.06 (3.9,4.22) |
| Barbados | 54.45 (-6.51,139.49) | 67.54 (-7.7,169.13) | 0.66 (0.62,0.71) | 3.41 (-0.35,9.73) | 4.4 (-0.46,12.49) | 0.75 (0.7,0.8) |
| Belarus | 32.11 (-3.08,87.71) | 41.54 (-4.79,108.09) | 0.91 (0.89,0.93) | 3.23 (-0.26,9.18) | 4.51 (-0.44,12.54) | 1.18 (1.15,1.22) |
| Belgium | 34.59 (-3.03,93.51) | 45.38 (-4.61,124.62) | 0.83 (0.78,0.89) | 4.26 (-0.31,12.46) | 6.2 (-0.56,17.94) | 1.16 (1.07,1.24) |
| Belize | 55.17 (-5.73,143.8) | 68.75 (-8.12,177.35) | 0.69 (0.61,0.76) | 3.37 (-0.3,9.6) | 4.54 (-0.47,12.67) | 0.9 (0.77,1.03) |
| Benin | 37.78 (-3.5,105.37) | 44.38 (-4.14,121.63) | 0.5 (0.46,0.54) | 2.67 (-0.2,7.74) | 3.45 (-0.27,9.85) | 0.84 (0.78,0.9) |
| Bermuda | 55.87 (-5.5,148.88) | 70.2 (-7.94,181.45) | 0.73 (0.7,0.75) | 3.83 (-0.35,11.05) | 4.72 (-0.46,13.34) | 0.7 (0.66,0.75) |
| Bhutan | 42.34 (-3.77,117.88) | 56.27 (-5.35,145.82) | 1.03 (1,1.06) | 3.11 (-0.24,8.86) | 5.07 (-0.44,14.36) | 1.72 (1.65,1.79) |
| Bolivia (Plurinational State of) | 43.37 (-4.21,115.46) | 60.54 (-6.72,157.39) | 1.12 (1.08,1.16) | 2.31 (-0.2,6.65) | 3.39 (-0.34,9.46) | 1.31 (1.26,1.36) |
| Bosnia and Herzegovina | 29.23 (-2.84,77.34) | 37.86 (-4.02,98.06) | 0.85 (0.79,0.91) | 3.12 (-0.26,9.19) | 4.41 (-0.39,12.15) | 1.18 (1.07,1.29) |
| Botswana | 31.83 (-2.7,90.11) | 44.19 (-4.24,115.26) | 0.98 (0.93,1.04) | 2.62 (-0.19,7.7) | 4.09 (-0.33,11.76) | 1.4 (1.34,1.46) |
| Brazil | 41.3 (-3.94,110.53) | 53.49 (-5.74,140.63) | 0.84 (0.82,0.86) | 2.8 (-0.24,7.88) | 3.83 (-0.34,10.65) | 1.06 (1.04,1.08) |
| Brunei Darussalam | 47.19 (-3.99,136.6) | 69.65 (-6.73,188.23) | 1.29 (1.15,1.43) | 2.52 (-0.18,7.87) | 4.29 (-0.34,12.32) | 1.81 (1.63,1.99) |
| Bulgaria | 32.77 (-3.15,88.03) | 37.71 (-3.55,97.82) | 0.45 (0.43,0.47) | 3.88 (-0.31,11.27) | 4.62 (-0.4,13.32) | 0.57 (0.53,0.6) |
| Burkina Faso | 19.22 (-1.5,56.94) | 26.23 (-2.2,75.84) | 1.02 (0.96,1.09) | 1.35 (-0.09,4.14) | 1.89 (-0.15,5.81) | 1.18 (1.1,1.25) |
| Burundi | 12.75 (-1.03,38.62) | 17.32 (-1.45,49.67) | 0.93 (0.86,1) | 0.99 (-0.07,3.22) | 1.33 (-0.09,4.36) | 0.88 (0.81,0.95) |
| Cabo Verde | 33.97 (-3.07,94.47) | 48.36 (-5.1,125.84) | 1.13 (1.12,1.14) | 2.43 (-0.2,7.48) | 3.82 (-0.32,11.5) | 0.86 (0.83,0.89) |
| Cambodia | 16.86 (-1.25,49.95) | 26 (-2.14,78.32) | 1.45 (1.41,1.5) | 0.85 (-0.06,2.67) | 1.26 (-0.08,3.8) | 1.52 (1.49,1.55) |
| Cameroon | 43.6 (-3.91,114.91) | 52.66 (-6.01,139.61) | 0.55 (0.54,0.57) | 3.42 (-0.29,9.83) | 4.29 (-0.39,12.2) | 1.36 (1.3,1.43) |
| Canada | 27.42 (-2.49,74.15) | 34.98 (-3.56,91.16) | 0.71 (0.66,0.76) | 3.76 (-0.32,10.76) | 5.55 (-0.56,16.02) | 0.7 (0.67,0.73) |
| Central African Republic | 19.34 (-1.55,57.37) | 29.29 (-2.27,82.81) | 1.37 (1.33,1.42) | 1.47 (-0.1,4.56) | 2.27 (-0.17,6.89) | 1.14 (1.05,1.24) |
| Chad | 21.55 (-1.64,63.04) | 26.45 (-1.96,75.72) | 0.58 (0.54,0.62) | 1.4 (-0.1,4.3) | 1.78 (-0.12,5.55) | 1.43 (1.38,1.48) |
| Chile | 52.01 (-5.39,135.08) | 65.99 (-7.94,167.25) | 0.77 (0.72,0.81) | 5.03 (-0.45,14.27) | 7.26 (-0.77,20.34) | 0.73 (0.69,0.78) |
| China | 36.73 (-3.15,106.16) | 69.32 (-6.53,188.43) | 2.47 (2.29,2.66) | 1.04 (-0.08,3.07) | 2.23 (-0.18,6.33) | 1.22 (1.13,1.3) |
| Colombia | 42.3 (-4.09,116.92) | 58.28 (-6.4,151.8) | 1.08 (1.06,1.1) | 2.34 (-0.21,6.66) | 3.36 (-0.33,9.62) | 2.63 (2.52,2.74) |
| Comoros | 22.32 (-1.53,66.61) | 33.78 (-3.27,90.92) | 1.4 (1.37,1.43) | 1.81 (-0.13,5.51) | 2.85 (-0.22,8.25) | 1.22 (1.2,1.25) |
| Congo | 28.25 (-2.33,80.88) | 41.19 (-3.92,112.62) | 1.2 (1.13,1.27) | 2.3 (-0.15,7.02) | 3.66 (-0.29,10.7) | 1.49 (1.46,1.52) |
| Cook Islands | 82.9 (-10.55,207.11) | 102.59 (-14.23,253.09) | 0.61 (0.56,0.67) | 4.5 (-0.47,12.5) | 5.66 (-0.75,15.06) | 1.48 (1.41,1.54) |
| Costa Rica | 48.56 (-5.34,130.86) | 61.36 (-7.14,163.98) | 0.78 (0.77,0.8) | 2.73 (-0.26,8.13) | 3.69 (-0.39,10.38) | 0.66 (0.56,0.76) |
| Croatia | 29.65 (-2.82,82.6) | 38.62 (-4.43,99.17) | 0.91 (0.87,0.96) | 3.36 (-0.28,9.88) | 4.69 (-0.41,13.32) | 0.95 (0.93,0.97) |
| Cuba | 45.48 (-4.23,123.83) | 61.77 (-7.17,161.12) | 1.04 (1.02,1.06) | 2.71 (-0.22,8.08) | 3.84 (-0.37,10.52) | 1.22 (1.15,1.28) |
| Cyprus | 30.85 (-2.64,89) | 45.28 (-4.62,120.49) | 1.29 (1.2,1.38) | 3.63 (-0.3,10.6) | 6.09 (-0.51,17.29) | 1.23 (1.19,1.28) |
| Czechia | 31.74 (-3.13,84.35) | 37.71 (-3.97,99.97) | 0.59 (0.58,0.61) | 3.65 (-0.29,10.43) | 4.65 (-0.45,12.89) | 1.72 (1.61,1.83) |
| Côte d'Ivoire | 36.04 (-3.24,97.37) | 44.62 (-4.26,118.75) | 0.68 (0.66,0.7) | 2.61 (-0.22,7.79) | 3.33 (-0.3,9.26) | 0.82 (0.8,0.84) |
| Democratic People's Republic of Korea | 22.88 (-2.01,69.13) | 32.92 (-2.66,98.21) | 1.13 (1.01,1.25) | 0.65 (-0.05,2.02) | 0.86 (-0.06,2.7) | 0.85 (0.72,0.97) |
| Democratic Republic of the Congo | 15.1 (-1.13,45.1) | 27.28 (-2.19,76.17) | 1.87 (1.79,1.95) | 1.19 (-0.08,3.82) | 2.15 (-0.16,6.41) | 1.81 (1.7,1.93) |
| Denmark | 31.37 (-2.61,87.78) | 41.52 (-4.18,113.06) | 0.92 (0.88,0.95) | 5.64 (-0.43,16.72) | 6.67 (-0.55,19.09) | 0.48 (0.32,0.65) |
| Djibouti | 15.77 (-1.28,44.57) | 23.67 (-1.88,67.82) | 1.3 (1.28,1.33) | 1.23 (-0.09,3.77) | 1.99 (-0.12,5.99) | 1.61 (1.58,1.64) |
| Dominica | 54.31 (-5.45,139.44) | 67.24 (-9.08,167.96) | 0.69 (0.66,0.72) | 3.27 (-0.3,9.39) | 4.18 (-0.45,11.75) | 0.81 (0.77,0.85) |
| Dominican Republic | 41.09 (-4.27,110.51) | 58.01 (-6.78,150.49) | 1.18 (1.14,1.22) | 2.4 (-0.19,7.1) | 3.61 (-0.36,10.12) | 1.47 (1.43,1.51) |
| Ecuador | 55.81 (-5.62,143.25) | 73.57 (-8.74,186.77) | 1.15 (1.05,1.25) | 3.33 (-0.27,9.25) | 4.46 (-0.44,12.4) | 0.93 (0.89,0.97) |
| Egypt | 47.34 (-5.3,123.03) | 63.51 (-8.93,158.33) | 0.96 (0.93,0.99) | 2.89 (-0.3,8.07) | 4.41 (-0.54,12.04) | 1.31 (1.27,1.36) |
| El Salvador | 49.97 (-5.42,134.03) | 63.97 (-7.09,161.53) | 0.81 (0.76,0.86) | 2.8 (-0.24,7.9) | 3.78 (-0.39,10.53) | 0.96 (0.89,1.03) |
| Equatorial Guinea | 27.47 (-2.33,74.98) | 44.25 (-4.58,115.48) | 1.61 (1.57,1.66) | 2.13 (-0.15,6.36) | 4.1 (-0.35,11.73) | 2.38 (2.28,2.48) |
| Eritrea | 11.49 (-1.02,34.87) | 16.58 (-1.27,53.06) | 1.06 (1.01,1.12) | 0.86 (-0.06,2.72) | 1.26 (-0.09,3.97) | 1.17 (1.1,1.23) |
| Estonia | 35.04 (-3.08,93.07) | 40.65 (-4.22,104.44) | 0.56 (0.53,0.58) | 3.68 (-0.29,10.76) | 4.64 (-0.4,13.6) | 0.89 (0.84,0.94) |
| Eswatini | 44.82 (-4.81,115.65) | 54.11 (-6.72,137.9) | 0.54 (0.48,0.59) | 3.96 (-0.4,11.2) | 5.1 (-0.53,14.05) | 0.69 (0.56,0.82) |
| Ethiopia | 15.14 (-1.02,43.96) | 16.74 (-1.45,46.68) | 0.19 (0.15,0.24) | 1.35 (-0.08,4.05) | 1.52 (-0.11,4.53) | 0.32 (0.28,0.36) |
| Fiji | 67.25 (-7.21,171.58) | 89.37 (-10.64,219.45) | 0.84 (0.8,0.88) | 3.37 (-0.27,9.96) | 4.53 (-0.49,12.51) | 0.87 (0.8,0.95) |
| Finland | 37.46 (-3.63,107.61) | 47.3 (-4.18,127.49) | 0.77 (0.72,0.82) | 4.82 (-0.38,14.22) | 6.7 (-0.54,18.81) | 1.08 (1,1.15) |
| France | 29.35 (-2.79,85.43) | 43.21 (-3.55,114.59) | 1.26 (1.16,1.36) | 3.86 (-0.32,11.49) | 6.4 (-0.52,18.48) | 1.69 (1.39,2) |
| Gabon | 39.15 (-4.17,104.7) | 53.49 (-6.21,136.32) | 0.98 (0.96,1) | 3.33 (-0.3,9.65) | 5.03 (-0.49,14.44) | 1.33 (1.28,1.37) |
| Gambia | 35.24 (-3.14,97.42) | 44.42 (-4.47,117.56) | 0.72 (0.7,0.74) | 2.44 (-0.19,7.22) | 3.34 (-0.26,10.01) | 1.03 (1,1.05) |
| Georgia | 28.32 (-2.92,77.08) | 31.62 (-3.34,82.53) | 0.4 (0.39,0.42) | 3.74 (-0.34,10.8) | 4.23 (-0.4,11.8) | 0.52 (0.47,0.56) |
| Germany | 39.43 (-3.43,106.46) | 46.68 (-4.77,126.32) | 0.53 (0.5,0.56) | 4.93 (-0.38,13.94) | 6.52 (-0.66,18.81) | 0.88 (0.82,0.94) |
| Ghana | 29.55 (-2.41,85.89) | 46.9 (-4.43,124.86) | 1.48 (1.41,1.55) | 2.09 (-0.16,6.37) | 3.73 (-0.31,10.47) | 1.88 (1.81,1.94) |
| Greece | 38.29 (-3.56,104.46) | 48.55 (-4.57,129.85) | 0.74 (0.69,0.8) | 4.34 (-0.36,12.53) | 6.11 (-0.53,17.36) | 1.63 (1.4,1.87) |
| Greenland | 32.35 (-2.94,88.73) | 38.87 (-3.9,101.72) | 0.64 (0.62,0.67) | 4.32 (-0.36,12.57) | 5.84 (-0.58,16.45) | 1 (0.94,1.07) |
| Grenada | 47.36 (-4.69,126.14) | 62.78 (-7.8,161.9) | 0.92 (0.88,0.96) | 2.73 (-0.22,7.79) | 3.82 (-0.43,10.75) | 1.13 (1.08,1.19) |
| Guam | 66.75 (-7.07,174.45) | 83.92 (-10.13,217.95) | 0.73 (0.71,0.75) | 3.56 (-0.37,10.21) | 4.47 (-0.47,12.14) | 0.78 (0.74,0.83) |
| Guatemala | 47.4 (-4.5,124.11) | 59.77 (-6.92,156.55) | 0.75 (0.74,0.76) | 2.51 (-0.19,7.27) | 3.3 (-0.33,9.28) | 0.93 (0.9,0.95) |
| Guinea | 28.9 (-2.45,81.71) | 35.93 (-3.06,96.32) | 0.7 (0.66,0.74) | 2.02 (-0.16,5.96) | 2.58 (-0.18,7.48) | 0.85 (0.77,0.93) |
| Guinea-Bissau | 29.55 (-2.62,84.12) | 37.19 (-3.34,101.88) | 0.73 (0.71,0.74) | 2.04 (-0.16,6.25) | 2.67 (-0.21,7.95) | 0.85 (0.82,0.88) |
| Guyana | 42.63 (-4.31,116.59) | 54.75 (-6.11,144.78) | 0.8 (0.78,0.83) | 2.52 (-0.21,7.57) | 3.33 (-0.32,9.2) | 0.98 (0.94,1.01) |
| Haiti | 24.45 (-2.04,67.12) | 37.8 (-3.16,105.44) | 1.54 (1.5,1.57) | 1.34 (-0.09,3.96) | 2.08 (-0.15,6.2) | 1.58 (1.52,1.63) |
| Honduras | 40.44 (-3.94,110.6) | 52.92 (-5.33,139.56) | 0.86 (0.81,0.92) | 2.11 (-0.19,6.1) | 2.94 (-0.23,8.31) | 1.07 (0.99,1.14) |
| Hungary | 34.07 (-3.29,90.9) | 40.71 (-3.98,105.52) | 0.61 (0.6,0.62) | 4.06 (-0.35,11.68) | 5.12 (-0.42,14.09) | 0.78 (0.75,0.82) |
| Iceland | 42.22 (-4.13,115.75) | 51.51 (-5.43,136.23) | 0.69 (0.67,0.72) | 6.7 (-0.6,18.94) | 7.99 (-0.74,22.84) | 0.44 (0.35,0.53) |
| India | 18.6 (-1.4,53.18) | 35.29 (-3.05,101.15) | 2.42 (2.25,2.58) | 1.35 (-0.09,3.96) | 2.92 (-0.23,8.25) | 2.96 (2.77,3.14) |
| Indonesia | 18.08 (-1.42,52.93) | 35.1 (-2.92,96.64) | 2.34 (2.21,2.47) | 1.12 (-0.07,3.3) | 2.14 (-0.15,6.01) | 2.33 (2.21,2.46) |
| Iran (Islamic Republic of) | 31.91 (-3.14,87.65) | 48.08 (-5.73,120.12) | 1.32 (1.29,1.34) | 2.1 (-0.17,5.79) | 3.49 (-0.34,9.3) | 1.61 (1.53,1.69) |
| Iraq | 45.2 (-4.77,119.16) | 53 (-5.79,133.41) | 0.55 (0.53,0.57) | 2.86 (-0.28,8.14) | 3.57 (-0.32,10.35) | 0.76 (0.71,0.81) |
| Ireland | 39.55 (-3.74,109.87) | 49.01 (-5.53,127.91) | 0.73 (0.7,0.76) | 4.97 (-0.41,14.75) | 6.9 (-0.65,19.25) | 1.05 (1,1.1) |
| Israel | 39.04 (-3.91,108.55) | 47.53 (-4.61,125.57) | 0.61 (0.57,0.64) | 4.59 (-0.39,13.48) | 6.15 (-0.55,17.34) | 0.87 (0.79,0.94) |
| Italy | 31.67 (-2.72,89.37) | 39.33 (-3.57,106.55) | 0.63 (0.59,0.66) | 4.18 (-0.32,12.01) | 5.65 (-0.45,15.63) | 0.98 (0.93,1.02) |
| Jamaica | 50.89 (-5.51,136.73) | 65.48 (-8.06,167.54) | 0.88 (0.84,0.93) | 3.04 (-0.25,8.65) | 4.08 (-0.44,11.27) | 1.05 (1,1.11) |
| Japan | 34.45 (-3.02,99.5) | 38.61 (-3.44,110.01) | 0.33 (0.28,0.37) | 2.08 (-0.15,5.92) | 2.58 (-0.18,7.51) | 0.69 (0.54,0.84) |
| Jordan | 47.21 (-5.6,121.29) | 63.37 (-8.48,159) | 0.96 (0.94,0.99) | 2.88 (-0.28,8.18) | 4.44 (-0.54,12.11) | 1.45 (1.39,1.5) |
| Kazakhstan | 29.01 (-2.84,77.28) | 35.38 (-4.09,92.22) | 0.63 (0.61,0.65) | 3.82 (-0.34,10.56) | 4.94 (-0.5,13.86) | 0.86 (0.83,0.88) |
| Kenya | 23.45 (-1.84,65.36) | 34.82 (-3.06,93.36) | 1.28 (1.25,1.31) | 2.18 (-0.15,6.37) | 3.34 (-0.27,9.19) | 1.41 (1.36,1.45) |
| Kiribati | 68.93 (-8.22,177.43) | 86.57 (-12.81,214.12) | 0.67 (0.59,0.74) | 3.39 (-0.32,9.59) | 4.19 (-0.55,11.65) | 0.58 (0.45,0.71) |
| Kuwait | 50.7 (-6.11,128.74) | 68.07 (-8.72,172.25) | 0.99 (0.96,1.02) | 3.38 (-0.35,9.54) | 5.11 (-0.57,13.89) | 1.44 (1.39,1.49) |
| Kyrgyzstan | 26.67 (-2.8,72.49) | 32.52 (-3.77,85.6) | 0.64 (0.61,0.66) | 3.32 (-0.31,9.5) | 4.17 (-0.45,11.68) | 0.73 (0.71,0.75) |
| Lao People's Democratic Republic | 18.04 (-1.32,53.77) | 32.76 (-2.45,94.46) | 2.23 (2.1,2.36) | 0.92 (-0.06,2.83) | 1.63 (-0.12,5.01) | 2.18 (2.05,2.3) |
| Latvia | 35.98 (-3.92,95.13) | 41.87 (-4.81,110.29) | 0.53 (0.51,0.54) | 3.78 (-0.33,10.8) | 4.72 (-0.5,13.89) | 0.76 (0.73,0.79) |
| Lebanon | 38.79 (-3.76,101.18) | 52.32 (-6.25,129.71) | 0.96 (0.94,0.98) | 2.44 (-0.22,6.97) | 3.78 (-0.36,10.72) | 1.32 (1.16,1.48) |
| Lesotho | 36.88 (-3.54,97.55) | 46.07 (-5.36,116.66) | 0.7 (0.69,0.72) | 2.93 (-0.21,8.5) | 3.96 (-0.41,10.75) | 0.96 (0.92,1) |
| Liberia | 38.09 (-3.69,100.82) | 48.01 (-5.05,126.64) | 0.81 (0.76,0.85) | 2.75 (-0.23,8.18) | 3.74 (-0.34,10.65) | 1.2 (1.08,1.31) |
| Libya | 44.33 (-5.02,116.78) | 61.08 (-8.56,158.03) | 1.08 (1.05,1.1) | 2.73 (-0.24,8.03) | 4.21 (-0.45,11.93) | 1.43 (1.39,1.48) |
| Lithuania | 31.45 (-3,86.48) | 39.1 (-4.09,102.73) | 0.73 (0.71,0.76) | 3.2 (-0.26,9.53) | 4.32 (-0.39,12.56) | 1 (0.93,1.07) |
| Luxembourg | 35.67 (-3.11,100.16) | 44.94 (-3.98,125.21) | 0.74 (0.72,0.76) | 4.51 (-0.37,13.62) | 6.32 (-0.5,17.98) | 1.03 (0.99,1.07) |
| Madagascar | 14.12 (-1.08,43.48) | 21.9 (-1.66,63.83) | 1.4 (1.31,1.48) | 1.09 (-0.07,3.43) | 1.68 (-0.12,5.34) | 1.39 (1.3,1.48) |
| Malawi | 19.78 (-1.48,57.02) | 31.42 (-2.79,86.01) | 1.52 (1.49,1.54) | 1.62 (-0.11,4.92) | 2.63 (-0.2,7.7) | 1.67 (1.63,1.7) |
| Malaysia | 37.54 (-3.56,105.87) | 55.58 (-5.45,142.66) | 1.32 (1.27,1.37) | 2.08 (-0.16,6.15) | 3.18 (-0.29,8.97) | 1.42 (1.35,1.49) |
| Maldives | 31.28 (-2.72,86.78) | 53.25 (-5.77,136.51) | 1.9 (1.84,1.97) | 1.62 (-0.12,4.88) | 2.88 (-0.28,8.02) | 2.1 (2.01,2.18) |
| Mali | 23.83 (-1.89,69.4) | 33.33 (-2.97,90.11) | 1.16 (1.07,1.24) | 1.61 (-0.11,4.86) | 2.4 (-0.19,7.04) | 1.45 (1.35,1.55) |
| Malta | 33.73 (-3,93.31) | 47.35 (-4.56,127.21) | 1.06 (0.95,1.17) | 4.16 (-0.32,12.12) | 6.62 (-0.59,19) | 1.44 (1.27,1.6) |
| Marshall Islands | 67.62 (-8.5,172.46) | 83.69 (-10.73,206.92) | 0.61 (0.57,0.64) | 3.14 (-0.33,8.69) | 3.89 (-0.39,10.78) | 0.6 (0.54,0.66) |
| Mauritania | 42.15 (-4.45,114.74) | 53.15 (-6.21,137.3) | 0.75 (0.73,0.77) | 3.19 (-0.26,9.52) | 4.36 (-0.43,12.37) | 0.97 (0.94,1) |
| Mauritius | 36.6 (-3.21,99.72) | 55.26 (-5.95,142.6) | 1.36 (1.31,1.41) | 2.06 (-0.13,6.1) | 3.11 (-0.29,8.91) | 1.4 (1.35,1.45) |
| Mexico | 50.42 (-5.25,131.48) | 61.41 (-7.33,153.72) | 0.62 (0.61,0.63) | 3.35 (-0.32,9.31) | 4.2 (-0.42,11.24) | 0.58 (0.5,0.66) |
| Micronesia (Federated States of) | 71.62 (-8.22,180.84) | 88.96 (-10.31,220.49) | 0.63 (0.56,0.69) | 3.61 (-0.35,10.27) | 4.41 (-0.48,11.95) | 0.52 (0.39,0.65) |
| Monaco | 42.92 (-3.91,116.38) | 50.46 (-5.3,132.68) | 0.54 (0.51,0.56) | 5.67 (-0.48,15.97) | 7.38 (-0.71,20.97) | 0.83 (0.78,0.87) |
| Mongolia | 23.74 (-2.36,64.24) | 28.94 (-3.01,76.26) | 0.56 (0.49,0.62) | 2.92 (-0.24,8.27) | 3.74 (-0.36,10.59) | 0.87 (0.83,0.92) |
| Montenegro | 31.29 (-2.66,84.96) | 39.91 (-4.38,102.87) | 0.82 (0.8,0.84) | 3.68 (-0.31,11.21) | 4.84 (-0.49,13.23) | 1.02 (0.98,1.05) |
| Morocco | 38.38 (-4.38,104.72) | 51.37 (-6.28,129.6) | 0.98 (0.97,0.99) | 2.18 (-0.18,6.37) | 3.29 (-0.36,9.06) | 1.35 (1.32,1.38) |
| Mozambique | 19.12 (-1.45,57.17) | 30.02 (-2.36,86.06) | 1.57 (1.51,1.62) | 1.5 (-0.1,4.96) | 2.43 (-0.17,7.28) | 1.72 (1.66,1.79) |
| Myanmar | 24.07 (-1.91,69.71) | 34.43 (-3.18,97.26) | 1.19 (1.14,1.24) | 1.22 (-0.08,3.71) | 1.74 (-0.13,5.09) | 1.21 (1.15,1.26) |
| Namibia | 29.56 (-2.54,85.79) | 40.62 (-3.75,104.85) | 0.98 (0.95,1.02) | 2.39 (-0.17,7.29) | 3.46 (-0.29,9.81) | 1.17 (1.1,1.24) |
| Nauru | 78.08 (-10.24,194.2) | 93.95 (-14.05,234.8) | 0.5 (0.47,0.54) | 4.09 (-0.43,11.33) | 4.78 (-0.62,13.23) | 0.42 (0.36,0.47) |
| Nepal | 19.98 (-1.44,61.02) | 38.27 (-3.25,107.74) | 2.41 (2.23,2.58) | 1.42 (-0.09,4.42) | 3.26 (-0.25,9.9) | 3.05 (2.87,3.24) |
| Netherlands | 37.31 (-3.64,104.31) | 48.42 (-4.8,131.16) | 0.88 (0.85,0.92) | 4.85 (-0.48,13.86) | 6.54 (-0.59,18.53) | 0.99 (0.94,1.03) |
| New Zealand | 44.12 (-4.34,121.39) | 56.64 (-5.67,150.17) | 0.84 (0.82,0.86) | 4.93 (-0.4,14.17) | 7.13 (-0.66,19.91) | 1.28 (1.23,1.32) |
| Nicaragua | 49.2 (-5.07,130.84) | 61.63 (-7.2,161.82) | 0.75 (0.71,0.79) | 2.66 (-0.22,7.42) | 3.51 (-0.35,9.92) | 0.9 (0.84,0.96) |
| Niger | 28.28 (-2.2,79.89) | 32.75 (-2.96,92.37) | 0.45 (0.44,0.46) | 1.92 (-0.13,6.02) | 2.24 (-0.18,6.68) | 0.52 (0.49,0.54) |
| Nigeria | 25.27 (-2.24,69.65) | 37.63 (-3.68,101.09) | 1.27 (1.11,1.43) | 2.07 (-0.16,5.94) | 3.07 (-0.27,8.59) | 1.11 (1,1.22) |
| Niue | 71.85 (-8.23,183.63) | 94.45 (-12.02,232.49) | 0.85 (0.79,0.91) | 3.78 (-0.34,10.92) | 5.07 (-0.57,13.6) | 0.91 (0.81,1.01) |
| North Macedonia | 30.63 (-3.08,80.85) | 38.52 (-3.91,101.36) | 0.79 (0.77,0.81) | 3.41 (-0.31,9.73) | 4.61 (-0.41,12.61) | 1.02 (0.99,1.05) |
| Northern Mariana Islands | 77.8 (-8.22,199.51) | 95.86 (-12.12,242.53) | 0.61 (0.53,0.68) | 4.22 (-0.43,12.03) | 5.1 (-0.6,13.81) | 0.44 (0.32,0.55) |
| Norway | 33.21 (-3.18,91.4) | 42.03 (-4.02,113.65) | 0.79 (0.76,0.81) | 4.63 (-0.37,13.02) | 6.5 (-0.56,18.12) | 1.16 (1.13,1.19) |
| Oman | 37.81 (-3.61,101.74) | 62.99 (-8.68,159.34) | 1.78 (1.74,1.82) | 2.2 (-0.17,6.62) | 4.41 (-0.54,12.82) | 2.49 (2.42,2.55) |
| Pakistan | 22.99 (-1.85,66.29) | 37.29 (-3.65,102.02) | 1.91 (1.79,2.04) | 2.49 (-0.19,7.05) | 4.6 (-0.39,12.84) | 2.36 (2.24,2.49) |
| Palau | 71.73 (-7.89,183.64) | 91.89 (-10.93,230.62) | 0.72 (0.65,0.78) | 3.78 (-0.35,10.09) | 4.95 (-0.54,13.7) | 0.72 (0.6,0.85) |
| Palestine | 45.24 (-5.05,117.45) | 56.87 (-7.93,141.66) | 0.71 (0.68,0.73) | 2.69 (-0.3,7.78) | 3.7 (-0.41,10.16) | 0.9 (0.84,0.96) |
| Panama | 37.23 (-3.27,104.65) | 53.8 (-4.97,142.21) | 1.17 (1.1,1.24) | 1.9 (-0.15,5.72) | 3.13 (-0.26,9.06) | 1.55 (1.47,1.63) |
| Papua New Guinea | 41.55 (-3.66,116.13) | 54.28 (-5.59,154.36) | 0.81 (0.74,0.87) | 1.83 (-0.15,5.5) | 2.32 (-0.17,6.92) | 0.7 (0.63,0.77) |
| Paraguay | 45.49 (-4.73,121.75) | 57.46 (-5.98,150.9) | 0.77 (0.76,0.79) | 2.67 (-0.23,7.98) | 3.52 (-0.32,9.78) | 0.94 (0.9,0.97) |
| Peru | 47.33 (-5.06,126.21) | 64.09 (-7.21,165.71) | 1.04 (1.02,1.06) | 2.58 (-0.21,7.57) | 3.68 (-0.35,10.47) | 1.22 (1.19,1.24) |
| Philippines | 20.89 (-1.66,59.81) | 32.28 (-2.77,89.14) | 1.35 (1.27,1.44) | 1.49 (-0.1,4.43) | 2.2 (-0.16,6.25) | 1.2 (1.14,1.25) |
| Poland | 27 (-2.56,74.2) | 34.83 (-3.45,92.28) | 0.85 (0.82,0.88) | 3.35 (-0.27,9.72) | 4.82 (-0.42,13.48) | 1.25 (1.21,1.29) |
| Portugal | 37.02 (-3.32,104.73) | 48.23 (-5.26,127) | 0.78 (0.71,0.85) | 4.36 (-0.39,12.62) | 6.35 (-0.58,17.95) | 0.97 (0.86,1.09) |
| Puerto Rico | 57.83 (-6.09,149.54) | 71.49 (-8.72,186.19) | 0.74 (0.7,0.78) | 3.95 (-0.36,11.38) | 5.04 (-0.5,13.8) | 0.89 (0.84,0.94) |
| Qatar | 49.65 (-4.98,127.63) | 68.5 (-9.52,168.12) | 1.06 (1.04,1.08) | 3.39 (-0.31,9.43) | 5.29 (-0.72,14.31) | 1.41 (1.35,1.47) |
| Republic of Korea | 33.07 (-3.25,99.71) | 50.66 (-5,149.01) | 1.55 (1.44,1.66) | 1.76 (-0.14,5.31) | 2.88 (-0.23,8.36) | 1.65 (1.63,1.67) |
| Republic of Moldova | 33.12 (-3.38,89.03) | 43.49 (-5.35,110.59) | 0.97 (0.94,1) | 3.24 (-0.32,9.36) | 4.75 (-0.49,13.28) | 1.36 (1.31,1.41) |
| Romania | 28.99 (-2.69,77.79) | 37.1 (-3.44,99.62) | 0.82 (0.78,0.85) | 3.24 (-0.27,9.65) | 4.58 (-0.4,12.73) | 1.14 (1.09,1.2) |
| Russian Federation | 31.65 (-3.25,84.03) | 39.93 (-4.72,101.04) | 0.8 (0.78,0.82) | 3.53 (-0.31,9.67) | 4.85 (-0.49,13.37) | 1.13 (1.1,1.16) |
| Rwanda | 17.69 (-1.35,51.04) | 26.25 (-2.48,75.07) | 1.22 (1.13,1.32) | 1.42 (-0.1,4.35) | 2.21 (-0.18,6.73) | 1.41 (1.3,1.52) |
| Saint Kitts and Nevis | 48.49 (-4.57,131.34) | 64.14 (-7.35,162.78) | 0.92 (0.9,0.94) | 3.03 (-0.23,8.84) | 4.15 (-0.38,11.68) | 1.06 (1.01,1.1) |
| Saint Lucia | 48.53 (-5.08,127.75) | 63.85 (-7.22,165.91) | 0.88 (0.85,0.92) | 2.94 (-0.26,8.5) | 3.98 (-0.4,11.13) | 1.01 (0.97,1.04) |
| Saint Vincent and the Grenadines | 39.54 (-3.61,109.4) | 55.27 (-5.19,148.06) | 1.15 (1.12,1.17) | 2.26 (-0.17,6.49) | 3.41 (-0.32,9.58) | 1.42 (1.37,1.47) |
| Samoa | 79.63 (-10.42,199.99) | 93.88 (-13.29,231.74) | 0.45 (0.4,0.5) | 4.21 (-0.45,11.51) | 4.8 (-0.56,13.17) | 0.33 (0.24,0.42) |
| San Marino | 40.47 (-3.5,112.4) | 48.61 (-4.83,129.15) | 0.6 (0.57,0.63) | 5.24 (-0.43,15.49) | 6.9 (-0.63,19.23) | 0.87 (0.81,0.93) |
| Sao Tome and Principe | 39.08 (-4.02,106.89) | 50.41 (-5.23,131.8) | 0.83 (0.81,0.84) | 2.83 (-0.22,8.4) | 3.98 (-0.35,11.19) | 1.13 (1.1,1.17) |
| Saudi Arabia | 44.06 (-4.56,114.49) | 65.51 (-8.08,162.78) | 1.31 (1.28,1.35) | 2.7 (-0.25,7.76) | 4.81 (-0.5,12.94) | 1.93 (1.88,1.98) |
| Senegal | 36.85 (-3.3,98.97) | 43.34 (-4.25,117.72) | 0.47 (0.45,0.49) | 2.66 (-0.22,7.82) | 3.23 (-0.25,9.44) | 0.57 (0.52,0.61) |
| Serbia | 31.41 (-2.82,84.54) | 41.3 (-4.35,107.52) | 0.93 (0.9,0.95) | 3.56 (-0.28,10.11) | 5.08 (-0.44,14.5) | 1.26 (1.22,1.3) |
| Seychelles | 49.55 (-5.43,133.45) | 66.54 (-8.3,171.56) | 0.93 (0.89,0.96) | 2.87 (-0.28,8.25) | 3.79 (-0.41,10.56) | 0.85 (0.82,0.88) |
| Sierra Leone | 27.82 (-2.13,79.59) | 36.27 (-3.38,100.91) | 0.83 (0.78,0.88) | 1.91 (-0.12,5.97) | 2.64 (-0.22,7.71) | 1.04 (0.93,1.15) |
| Singapore | 36.42 (-3.26,107.07) | 64.11 (-5.81,179.7) | 1.7 (1.59,1.82) | 1.95 (-0.14,5.81) | 3.91 (-0.29,11.95) | 2.19 (2.05,2.33) |
| Slovakia | 32.51 (-3.19,86.47) | 38.28 (-3.98,98.95) | 0.51 (0.49,0.52) | 3.7 (-0.31,10.92) | 4.72 (-0.47,13.51) | 0.75 (0.72,0.79) |
| Slovenia | 31.3 (-2.91,87.12) | 38.72 (-3.88,102.95) | 0.73 (0.71,0.75) | 3.62 (-0.27,10.39) | 4.82 (-0.4,13.61) | 0.99 (0.93,1.05) |
| Solomon Islands | 56.79 (-5.26,153.6) | 71.19 (-7.55,181.01) | 0.65 (0.58,0.72) | 2.62 (-0.23,7.64) | 3.22 (-0.3,9.02) | 0.56 (0.46,0.67) |
| Somalia | 25.2 (-1.96,74.39) | 32.25 (-2.84,88.51) | 0.85 (0.83,0.88) | 1.96 (-0.13,5.96) | 2.52 (-0.19,7.41) | 0.9 (0.86,0.94) |
| South Africa | 39.49 (-4.16,101.26) | 47.86 (-6.04,119.09) | 0.62 (0.61,0.63) | 3.7 (-0.32,9.98) | 4.51 (-0.5,12.24) | 0.69 (0.67,0.72) |
| South Sudan | 15.91 (-1.17,47.19) | 19.27 (-1.57,56.16) | 0.61 (0.59,0.63) | 1.25 (-0.09,3.86) | 1.55 (-0.11,4.71) | 0.69 (0.66,0.73) |
| Spain | 41.88 (-3.77,114.74) | 51.19 (-4.95,139.17) | 0.59 (0.56,0.62) | 5.3 (-0.46,15.36) | 7.11 (-0.68,19.8) | 0.82 (0.71,0.94) |
| Sri Lanka | 25.73 (-2.12,77.09) | 39.03 (-3.3,111.36) | 1.33 (1.26,1.4) | 1.35 (-0.09,4.02) | 2.04 (-0.14,6.14) | 1.33 (1.25,1.4) |
| Sudan | 35.24 (-3.56,93.71) | 51.8 (-5.97,132.43) | 1.27 (1.23,1.31) | 1.89 (-0.17,5.52) | 3.28 (-0.33,9.43) | 1.81 (1.74,1.88) |
| Suriname | 38.54 (-3.6,110.33) | 53.9 (-5.77,142.76) | 1.1 (1.07,1.12) | 2.3 (-0.19,6.97) | 3.41 (-0.29,9.88) | 1.32 (1.29,1.35) |
| Sweden | 26.14 (-2.64,74.58) | 34.81 (-3.27,93.35) | 0.97 (0.91,1.03) | 3.48 (-0.31,10.36) | 6.02 (-0.5,17.09) | 1.86 (1.63,2.09) |
| Switzerland | 32.13 (-2.79,90.32) | 39.55 (-3.71,107.25) | 0.7 (0.68,0.71) | 3.98 (-0.3,11.8) | 5.28 (-0.43,15.64) | 0.95 (0.92,0.97) |
| Syrian Arab Republic | 41.99 (-4.48,111.6) | 58.07 (-7.87,145.4) | 1.07 (1.04,1.1) | 2.49 (-0.22,7) | 3.79 (-0.47,10.56) | 1.33 (1.28,1.39) |
| Taiwan (Province of China) | 41.58 (-3.54,123.5) | 80.33 (-8.22,225.49) | 2.2 (2.13,2.27) | 1.25 (-0.1,3.9) | 2.44 (-0.21,7.07) | 2.31 (2.23,2.4) |
| Tajikistan | 24.83 (-2.39,67.01) | 29.3 (-3.39,76.36) | 0.57 (0.55,0.58) | 2.89 (-0.27,8.23) | 3.46 (-0.34,9.37) | 0.62 (0.6,0.64) |
| Thailand | 29.74 (-2.73,83.83) | 55.94 (-5.74,153.91) | 2.1 (2.03,2.18) | 1.48 (-0.09,4.45) | 2.86 (-0.26,8.4) | 2.23 (2.16,2.3) |
| Timor-Leste | 11.4 (-0.9,35.4) | 20.44 (-1.38,59.58) | 1.95 (1.87,2.02) | 0.61 (-0.04,1.89) | 1.03 (-0.07,3.32) | 1.77 (1.69,1.84) |
| Togo | 30.63 (-2.86,84.13) | 40.98 (-3.77,109.26) | 0.92 (0.9,0.94) | 2.12 (-0.15,6.4) | 3.01 (-0.21,8.92) | 1.14 (1.1,1.18) |
| Tokelau | 65 (-7.58,165.47) | 87.55 (-10.45,212.99) | 0.93 (0.88,0.97) | 3.19 (-0.32,9.03) | 4.53 (-0.52,12.64) | 1.06 (0.98,1.14) |
| Tonga | 82.26 (-10.38,203.22) | 99 (-13.55,243.81) | 0.5 (0.42,0.58) | 4.21 (-0.44,11.63) | 5.12 (-0.66,13.59) | 0.39 (0.23,0.56) |
| Trinidad and Tobago | 51.38 (-5.11,137.66) | 65.08 (-8.26,167.11) | 0.83 (0.81,0.85) | 3.23 (-0.28,9.2) | 4.16 (-0.43,11.79) | 0.98 (0.92,1.04) |
| Tunisia | 36.86 (-3.63,101.26) | 52.81 (-6.54,132.05) | 1.22 (1.19,1.26) | 2.13 (-0.19,6.23) | 3.45 (-0.37,9.47) | 1.59 (1.57,1.62) |
| Türkiye | 46.75 (-5.06,122.26) | 62.18 (-7.74,157.09) | 0.89 (0.87,0.91) | 2.66 (-0.27,7.43) | 4.01 (-0.43,11.18) | 1.25 (1.21,1.29) |
| Turkmenistan | 25.06 (-2.29,68.11) | 30.52 (-3.11,82.77) | 0.65 (0.64,0.67) | 3.13 (-0.25,9.15) | 4.14 (-0.36,11.81) | 0.93 (0.91,0.95) |
| Tuvalu | 60.01 (-5.93,159.2) | 82.71 (-10.19,206.75) | 1 (0.97,1.04) | 2.92 (-0.24,8.58) | 4.13 (-0.41,11.23) | 1.03 (0.95,1.1) |
| Uganda | 20.51 (-1.47,62.34) | 31.57 (-3.01,86.48) | 1.54 (1.5,1.59) | 1.6 (-0.12,4.96) | 2.57 (-0.23,7.62) | 1.72 (1.66,1.77) |
| Ukraine | 31.79 (-3.08,85.66) | 37.7 (-4.09,97.22) | 0.59 (0.56,0.62) | 3.61 (-0.28,10.06) | 4.44 (-0.45,12.82) | 0.75 (0.7,0.8) |
| United Arab Emirates | 43.7 (-4.61,114.75) | 66.87 (-8.96,168.21) | 1.42 (1.39,1.45) | 2.74 (-0.23,7.53) | 4.9 (-0.59,13.58) | 1.99 (1.94,2.05) |
| United Kingdom | 40.42 (-3.82,110.13) | 51.34 (-5.24,135.05) | 0.82 (0.73,0.91) | 5.53 (-0.47,15.32) | 7.74 (-0.71,21.2) | 1.09 (1.01,1.17) |
| United Republic of Tanzania | 26.76 (-2.26,77.04) | 38.77 (-3.31,105.38) | 1.27 (1.2,1.33) | 2.12 (-0.15,6.14) | 3.28 (-0.23,9.47) | 1.51 (1.45,1.58) |
| United States of America | 57.68 (-5.66,154.68) | 70.11 (-8.17,178.36) | 0.71 (0.55,0.87) | 7.69 (-0.67,21.29) | 9.98 (-1.01,27.1) | 1.15 (1.02,1.29) |
| United States Virgin Islands | 59.8 (-6.57,156.67) | 71.52 (-7.98,182.19) | 0.61 (0.59,0.63) | 4.02 (-0.39,11.3) | 4.93 (-0.47,13.46) | 0.7 (0.66,0.73) |
| Uruguay | 45.09 (-4.36,126.37) | 57.43 (-6.07,149.76) | 0.78 (0.75,0.81) | 4.19 (-0.35,12.17) | 6.11 (-0.57,17.25) | 1.3 (1.22,1.39) |
| Uzbekistan | 26.19 (-2.81,70) | 32.95 (-3.7,86.5) | 0.77 (0.76,0.78) | 3.24 (-0.27,9.63) | 4.33 (-0.43,11.84) | 1.02 (0.99,1.04) |
| Vanuatu | 52.49 (-4.86,141.09) | 68.14 (-7.23,173.13) | 0.8 (0.76,0.84) | 2.5 (-0.2,7.25) | 3.18 (-0.35,8.79) | 0.75 (0.71,0.8) |
| Venezuela (Bolivarian Republic of) | 50.72 (-5.26,135.88) | 63.07 (-7.35,162.75) | 0.72 (0.71,0.73) | 2.88 (-0.26,8.06) | 3.72 (-0.41,10.34) | 0.84 (0.81,0.87) |
| Viet Nam | 9.94 (-0.8,30.52) | 22.93 (-1.89,67.33) | 3.19 (3,3.38) | 0.5 (-0.04,1.56) | 1.1 (-0.07,3.44) | 3.06 (2.87,3.26) |
| Yemen | 26.17 (-2.04,73.88) | 36.04 (-3.74,98.14) | 1.08 (1.03,1.14) | 1.36 (-0.09,4.1) | 2.04 (-0.16,5.95) | 1.4 (1.33,1.47) |
| Zambia | 21.02 (-1.65,59.37) | 35.17 (-3.12,93.76) | 1.7 (1.68,1.72) | 1.71 (-0.13,5.33) | 2.97 (-0.24,9.01) | 1.76 (1.73,1.8) |
| Zimbabwe | 31.04 (-2.69,86.82) | 41.36 (-3.78,106.53) | 0.86 (0.81,0.91) | 2.54 (-0.18,7.72) | 3.37 (-0.28,9.63) | 0.81 (0.73,0.89) |
