# Supplementary material for: Global, regional, and national burden of early-onset OA attributable to high BMI: 1990–2021 estimates and 2036 projections from the global burden of disease study
Source: PLoS One. 2025 Jul 16;20(7):e0328414. doi: 10.1371/journal.pone.0328414 (PMC12266449; doi:10.1371/journal.pone.0328414)
Supplement: S3 Table — (DOCX) [file pone.0328414.s010.docx]

| Table S3. The ASDR of early-onset osteoarthritis attributed to high BMI in 1990 and 2021 for both sexes by countries, with EAPC from 1990 to 2021. | | | | | | |
| --- | --- | --- | --- | --- | --- | --- |
|  | **Knee OA** | | | **Hip OA** | | |
| **Location** | **ASDR in 1990 (per 100,000)** | **ASDR in 2021 (per 100,000)** | **EAPC of ASDR (%) 1990–2021** | **ASDR in 1990 (per 100,000)** | **ASDR in 2021 (per 100,000)** | **EAPC of ASDR (%) 1990–2021** |
| **Country** | | | | | | |
| Afghanistan | 26.59 (-2.27,72.97) | 34.27 (-3.26,92.1) | 0.9 (0.86,0.94) | 1.61 (-0.12,4.66) | 2.35 (-0.2,6.49) | 1.35 (1.24,1.46) |
| Albania | 25.78 (-2.46,69.2) | 33.03 (-3.77,86.24) | 0.84 (0.82,0.85) | 3.31 (-0.29,9.23) | 4.4 (-0.43,12.32) | 1 (0.97,1.02) |
| Algeria | 29.54 (-2.65,82.89) | 46.26 (-5.06,119.45) | 1.51 (1.47,1.55) | 1.97 (-0.17,5.55) | 3.62 (-0.34,9.91) | 2.05 (2.02,2.08) |
| American Samoa | 71.82 (-9.32,178.56) | 84.51 (-11.54,206.68) | 0.45 (0.36,0.55) | 5.49 (-0.6,14.94) | 6.35 (-0.81,16.72) | 0.26 (0.1,0.42) |
| Andorra | 32.28 (-3.26,89.95) | 39.75 (-3.93,107.63) | 0.71 (0.69,0.72) | 5.14 (-0.46,14.98) | 6.88 (-0.57,19.44) | 0.93 (0.89,0.98) |
| Angola | 15.71 (-1.22,45.79) | 27.55 (-2.31,77.21) | 1.82 (1.79,1.85) | 1.36 (-0.09,4.07) | 2.58 (-0.19,7.54) | 2.06 (2.01,2.11) |
| Antigua and Barbuda | 39.45 (-4.18,106.56) | 52.78 (-6.08,136.73) | 0.95 (0.93,0.97) | 2.89 (-0.27,8.16) | 4.01 (-0.37,11.14) | 1.08 (1.07,1.1) |
| Argentina | 39.83 (-4.07,107.8) | 53.38 (-5.92,134.48) | 0.98 (0.92,1.04) | 4.71 (-0.47,13.22) | 7.36 (-0.76,20.02) | 1.48 (1.37,1.59) |
| Armenia | 24.24 (-2.44,65.67) | 29.7 (-3.39,76.72) | 0.69 (0.67,0.7) | 3.45 (-0.29,9.77) | 4.59 (-0.47,12.9) | 1 (0.97,1.03) |
| Australia | 41.02 (-4.15,111.66) | 57.54 (-6.38,151.59) | 1.09 (1.03,1.15) | 5.35 (-0.5,14.93) | 8.66 (-0.85,23.89) | 1.59 (1.49,1.7) |
| Austria | 30.4 (-2.92,84.87) | 37.79 (-3.46,103.96) | 0.68 (0.67,0.7) | 4.65 (-0.4,13.54) | 6.36 (-0.56,18.17) | 0.97 (0.94,0.99) |
| Azerbaijan | 24.81 (-2.58,65.91) | 30.72 (-3.34,78.48) | 0.77 (0.74,0.79) | 3.61 (-0.33,10.04) | 4.83 (-0.46,13.35) | 1.06 (1,1.11) |
| Bahamas | 44.96 (-4.46,121.33) | 57.67 (-6.55,147.7) | 0.82 (0.79,0.86) | 3.56 (-0.31,9.94) | 4.58 (-0.44,12.87) | 0.82 (0.79,0.86) |
| Bahrain | 39 (-3.86,105.17) | 54.52 (-7.25,137.99) | 1.11 (1.1,1.12) | 3.15 (-0.27,8.72) | 4.93 (-0.6,13.36) | 1.44 (1.41,1.47) |
| Bangladesh | 10.71 (-0.81,32.19) | 26.88 (-2.23,75.95) | 3.48 (3.33,3.63) | 0.72 (-0.05,2.21) | 2.14 (-0.17,6.29) | 4.11 (3.94,4.29) |
| Barbados | 45.36 (-5.03,119.64) | 58.22 (-6.25,147.79) | 0.79 (0.76,0.83) | 3.48 (-0.32,9.81) | 4.63 (-0.44,12.78) | 0.89 (0.85,0.94) |
| Belarus | 29.15 (-2.75,80.79) | 38.3 (-4.4,99.11) | 0.96 (0.93,0.98) | 3.54 (-0.28,9.99) | 4.94 (-0.51,13.66) | 1.17 (1.15,1.19) |
| Belgium | 29.36 (-2.52,79.83) | 38.81 (-3.84,106.8) | 0.87 (0.82,0.91) | 4.46 (-0.34,12.59) | 6.45 (-0.59,18.07) | 1.15 (1.08,1.22) |
| Belize | 45.69 (-4.58,120.26) | 59.53 (-6.79,154.04) | 0.83 (0.75,0.91) | 3.52 (-0.32,9.89) | 4.84 (-0.49,13.27) | 0.97 (0.83,1.12) |
| Benin | 28.52 (-2.48,79.77) | 38.26 (-3.44,104.99) | 0.92 (0.89,0.96) | 2.22 (-0.16,6.29) | 3.38 (-0.27,9.77) | 1.35 (1.28,1.41) |
| Bermuda | 49.03 (-5.17,129.76) | 62.2 (-7.38,159.3) | 0.76 (0.73,0.8) | 4.22 (-0.41,11.89) | 5.27 (-0.55,14.68) | 0.73 (0.69,0.77) |
| Bhutan | 32.01 (-2.88,87.79) | 45.71 (-4.31,121.3) | 1.23 (1.18,1.28) | 2.25 (-0.17,6.5) | 3.88 (-0.35,11.07) | 1.9 (1.82,1.98) |
| Bolivia (Plurinational State of) | 35.53 (-3.45,95.43) | 50.88 (-5.31,131.57) | 1.2 (1.16,1.24) | 2.4 (-0.2,6.83) | 3.68 (-0.36,9.82) | 1.47 (1.42,1.51) |
| Bosnia and Herzegovina | 26.73 (-2.74,72.86) | 33.89 (-3.64,89.95) | 0.78 (0.72,0.84) | 3.36 (-0.29,9.61) | 4.6 (-0.42,12.53) | 1.06 (0.95,1.17) |
| Botswana | 22.27 (-1.79,63.02) | 39.33 (-3.57,105.79) | 1.85 (1.84,1.87) | 2.07 (-0.14,6.27) | 4.48 (-0.34,12.61) | 2.53 (2.46,2.6) |
| Brazil | 35.5 (-3.37,96.03) | 47.88 (-5.21,125.28) | 0.97 (0.96,0.98) | 2.99 (-0.25,8.26) | 4.28 (-0.4,11.87) | 1.21 (1.19,1.23) |
| Brunei Darussalam | 37.67 (-3.16,108) | 56.5 (-5.51,153.23) | 1.37 (1.24,1.5) | 3 (-0.22,8.94) | 4.88 (-0.39,13.9) | 1.7 (1.56,1.84) |
| Bulgaria | 30.22 (-3,82.1) | 34.34 (-3.25,88.54) | 0.41 (0.4,0.43) | 4.2 (-0.36,11.86) | 4.92 (-0.44,13.71) | 0.5 (0.47,0.52) |
| Burkina Faso | 17.12 (-1.34,49.62) | 24.09 (-1.99,68.35) | 1.12 (1.08,1.16) | 1.36 (-0.09,4.15) | 2 (-0.15,6.09) | 1.3 (1.26,1.34) |
| Burundi | 10.45 (-0.85,31.85) | 14.37 (-1.19,41.16) | 0.99 (0.9,1.08) | 0.91 (-0.06,2.86) | 1.29 (-0.08,4.07) | 1.07 (0.98,1.16) |
| Cabo Verde | 27.88 (-2.55,77.21) | 40.66 (-3.9,109.21) | 1.21 (1.2,1.22) | 2.18 (-0.17,6.65) | 3.69 (-0.29,10.72) | 1.2 (1.17,1.22) |
| Cambodia | 13.13 (-0.98,38.56) | 20.59 (-1.66,62.27) | 1.49 (1.47,1.51) | 0.79 (-0.05,2.41) | 1.25 (-0.09,3.69) | 1.74 (1.71,1.77) |
| Cameroon | 35.8 (-3.15,96.32) | 47.17 (-5.07,121.24) | 0.86 (0.85,0.88) | 3.16 (-0.25,9.15) | 4.45 (-0.41,12.3) | 1.58 (1.52,1.63) |
| Canada | 24.33 (-2.33,65.86) | 31.66 (-3.45,83.1) | 0.87 (0.79,0.94) | 4.17 (-0.36,11.74) | 6.31 (-0.62,17.59) | 1.05 (1.02,1.07) |
| Central African Republic | 14.38 (-1.13,42.03) | 23.46 (-1.86,66.1) | 1.63 (1.58,1.68) | 1.21 (-0.08,3.62) | 2.07 (-0.15,6.24) | 1.27 (1.16,1.38) |
| Chad | 17.44 (-1.4,50.98) | 22.59 (-1.76,64.1) | 0.76 (0.72,0.8) | 1.27 (-0.09,3.87) | 1.75 (-0.13,5.37) | 1.82 (1.78,1.86) |
| Chile | 44.47 (-4.67,117.21) | 57.95 (-7.06,146.04) | 0.85 (0.8,0.9) | 5.47 (-0.52,15.05) | 8.32 (-0.94,22.77) | 0.98 (0.95,1.01) |
| China | 27.68 (-2.34,80.15) | 52.98 (-4.9,144.88) | 2.55 (2.35,2.75) | 1.1 (-0.08,3.22) | 2.4 (-0.2,6.75) | 1.35 (1.24,1.46) |
| Colombia | 35.39 (-3.26,97.84) | 50.78 (-5.52,134.1) | 1.18 (1.16,1.21) | 2.44 (-0.19,7.05) | 3.58 (-0.35,10.02) | 2.66 (2.58,2.73) |
| Comoros | 17.61 (-1.29,52.44) | 30.14 (-2.73,81.63) | 1.78 (1.75,1.81) | 1.63 (-0.12,4.9) | 2.98 (-0.22,8.39) | 1.26 (1.23,1.28) |
| Congo | 22.54 (-1.97,64.35) | 34.56 (-3.35,95.02) | 1.35 (1.3,1.4) | 2.09 (-0.15,6.15) | 3.55 (-0.29,10.16) | 2.01 (1.99,2.03) |
| Cook Islands | 68.72 (-8.39,170.9) | 85.73 (-11.97,211.54) | 0.65 (0.6,0.7) | 5.02 (-0.53,13.4) | 6.3 (-0.77,16.56) | 1.72 (1.67,1.76) |
| Costa Rica | 41.25 (-4.53,111.82) | 54.15 (-6.19,142.47) | 0.89 (0.88,0.91) | 2.9 (-0.27,8.46) | 3.95 (-0.41,10.94) | 0.62 (0.5,0.73) |
| Croatia | 29 (-2.9,79.6) | 36.46 (-4.26,93.65) | 0.79 (0.76,0.83) | 3.93 (-0.33,11.06) | 5.22 (-0.5,14.57) | 0.99 (0.96,1.01) |
| Cuba | 37.26 (-3.35,103.97) | 52.68 (-5.85,138) | 1.16 (1.14,1.19) | 2.75 (-0.22,8.08) | 4.07 (-0.41,11.2) | 1 (0.95,1.06) |
| Cyprus | 27.26 (-2.45,76.2) | 39.85 (-4.37,105.95) | 1.28 (1.21,1.35) | 4 (-0.33,11.51) | 6.42 (-0.55,18.09) | 1.37 (1.33,1.41) |
| Czechia | 30.96 (-3.11,81.3) | 35.95 (-3.77,95.81) | 0.5 (0.49,0.51) | 4.23 (-0.37,11.63) | 5.2 (-0.5,14.31) | 1.6 (1.51,1.69) |
| C么te d'Ivoire | 28.27 (-2.42,80.22) | 38.6 (-3.57,105.04) | 0.99 (0.96,1.01) | 2.32 (-0.17,6.79) | 3.35 (-0.27,9.46) | 0.67 (0.66,0.69) |
| Democratic People's Republic of Korea | 18.23 (-1.63,54.76) | 25.15 (-2.05,73.86) | 1 (0.89,1.1) | 0.69 (-0.05,2.07) | 0.9 (-0.06,2.67) | 0.77 (0.66,0.88) |
| Democratic Republic of the Congo | 13.96 (-1.05,40.62) | 25.88 (-2.08,72.89) | 1.97 (1.89,2.05) | 1.26 (-0.08,3.91) | 2.39 (-0.16,7.07) | 2 (1.88,2.11) |
| Denmark | 28.65 (-2.57,78.98) | 37.49 (-4.01,102.04) | 0.88 (0.85,0.91) | 6.26 (-0.5,18.62) | 7.03 (-0.69,19.75) | 0.33 (0.14,0.51) |
| Djibouti | 11.94 (-0.98,33.9) | 19.51 (-1.46,55.76) | 1.6 (1.54,1.65) | 1.06 (-0.08,3.17) | 1.95 (-0.12,5.65) | 2.04 (1.99,2.1) |
| Dominica | 44.2 (-4.26,114.81) | 56.5 (-6.98,144.25) | 0.81 (0.8,0.82) | 3.21 (-0.28,9.29) | 4.35 (-0.46,11.99) | 0.99 (0.94,1.04) |
| Dominican Republic | 34.32 (-3.48,91.91) | 49.98 (-5.53,130.18) | 1.26 (1.22,1.31) | 2.49 (-0.2,7.37) | 3.88 (-0.39,10.82) | 1.57 (1.52,1.63) |
| Ecuador | 43.78 (-4.35,115.32) | 61.37 (-6.92,158.38) | 1.24 (1.17,1.31) | 3.26 (-0.26,8.98) | 4.74 (-0.49,12.9) | 1.22 (1.17,1.26) |
| Egypt | 39.22 (-4.16,103.71) | 55.3 (-6.93,141.05) | 1.09 (1.06,1.12) | 2.78 (-0.28,7.88) | 4.48 (-0.51,11.92) | 1.43 (1.36,1.49) |
| El Salvador | 42.97 (-4.85,113.84) | 56.99 (-6.35,143.43) | 0.91 (0.86,0.96) | 2.96 (-0.27,8.4) | 4.02 (-0.39,10.99) | 0.96 (0.88,1.03) |
| Equatorial Guinea | 22.47 (-1.84,62.98) | 40.12 (-3.99,106.38) | 1.97 (1.89,2.04) | 1.97 (-0.14,6.05) | 4.3 (-0.38,12.11) | 2.82 (2.7,2.94) |
| Eritrea | 8.31 (-0.71,24.82) | 14.38 (-1.06,45.14) | 1.71 (1.68,1.74) | 0.69 (-0.05,2.16) | 1.28 (-0.09,3.87) | 2.02 (1.98,2.06) |
| Estonia | 32.49 (-2.83,88.46) | 38.75 (-4.27,101) | 0.65 (0.62,0.68) | 4.12 (-0.33,11.74) | 5.29 (-0.52,15.02) | 0.96 (0.92,1.01) |
| Eswatini | 34.17 (-3.41,89.69) | 47.02 (-5.33,123.41) | 0.96 (0.87,1.06) | 3.58 (-0.32,10.34) | 5.5 (-0.54,14.78) | 1.24 (1.07,1.4) |
| Ethiopia | 12.44 (-0.85,35.76) | 16.81 (-1.46,46.41) | 0.94 (0.92,0.97) | 1.26 (-0.08,3.72) | 1.85 (-0.14,5.34) | 1.25 (1.23,1.27) |
| Fiji | 53.13 (-5.46,137.89) | 70.95 (-8.11,178.75) | 0.86 (0.82,0.91) | 3.43 (-0.27,9.72) | 4.77 (-0.47,13.04) | 0.98 (0.89,1.07) |
| Finland | 32.35 (-3.21,89.99) | 41.02 (-4.03,108.73) | 0.77 (0.72,0.82) | 5.08 (-0.44,14.52) | 7 (-0.61,19.26) | 1.04 (0.97,1.11) |
| France | 26.48 (-2.64,76.07) | 37.88 (-3.36,101.87) | 1.17 (1.09,1.24) | 4.32 (-0.37,12.17) | 6.72 (-0.54,19.34) | 1.47 (1.22,1.72) |
| Gabon | 30.65 (-3.02,84.23) | 45.94 (-5.03,120.37) | 1.32 (1.31,1.34) | 2.92 (-0.25,8.4) | 5.02 (-0.51,14.22) | 1.75 (1.69,1.81) |
| Gambia | 26.83 (-2.31,73.74) | 37.32 (-3.53,99.99) | 1.06 (1.02,1.1) | 2.16 (-0.17,6.26) | 3.21 (-0.25,9.31) | 1.27 (1.22,1.32) |
| Georgia | 26.14 (-2.55,70.94) | 29.13 (-3.08,75.29) | 0.4 (0.39,0.41) | 4.09 (-0.35,11.43) | 4.61 (-0.43,12.82) | 0.48 (0.46,0.5) |
| Germany | 34.38 (-3.17,92.73) | 40.8 (-4.4,108.67) | 0.54 (0.51,0.57) | 5.38 (-0.47,15.01) | 6.95 (-0.72,19.43) | 0.82 (0.77,0.86) |
| Ghana | 23.32 (-1.96,65.28) | 38.23 (-3.53,101.7) | 1.57 (1.55,1.6) | 1.86 (-0.13,5.53) | 3.39 (-0.27,9.55) | 1.9 (1.87,1.94) |
| Greece | 33.23 (-3.18,89.74) | 43.02 (-4.62,114.11) | 0.8 (0.74,0.87) | 4.66 (-0.41,13.43) | 6.54 (-0.62,18.34) | 1.75 (1.47,2.02) |
| Greenland | 27.24 (-2.72,73.81) | 34.01 (-3.44,89.5) | 0.78 (0.75,0.81) | 4.73 (-0.46,13.34) | 6.48 (-0.61,17.46) | 1.08 (1.02,1.14) |
| Grenada | 36.63 (-3.52,97.73) | 51.06 (-5.81,133.73) | 1.08 (1.05,1.1) | 2.53 (-0.2,7.3) | 3.82 (-0.39,10.48) | 1.36 (1.31,1.42) |
| Guam | 57.19 (-5.92,147.5) | 70.64 (-8.24,181.44) | 0.67 (0.64,0.7) | 4.17 (-0.45,11.53) | 5.07 (-0.5,13.85) | 0.65 (0.61,0.69) |
| Guatemala | 39.56 (-3.61,107.29) | 52.4 (-5.97,137.79) | 0.91 (0.9,0.91) | 2.58 (-0.2,7.43) | 3.53 (-0.36,9.83) | 1.04 (1.01,1.06) |
| Guinea | 21.54 (-1.83,61.69) | 29.53 (-2.5,79.76) | 1 (0.95,1.05) | 1.67 (-0.12,5.03) | 2.36 (-0.16,6.75) | 1.17 (1.11,1.23) |
| Guinea-Bissau | 22.73 (-1.86,64.44) | 31.43 (-2.68,88.36) | 1.02 (1,1.04) | 1.73 (-0.12,5.14) | 2.54 (-0.2,7.37) | 1.19 (1.16,1.21) |
| Guyana | 35.52 (-3.4,98.13) | 47.33 (-4.98,125.58) | 0.93 (0.91,0.95) | 2.62 (-0.21,7.59) | 3.59 (-0.35,9.97) | 1.07 (1.03,1.1) |
| Haiti | 17.99 (-1.52,49.63) | 29.73 (-2.47,84.16) | 1.75 (1.72,1.78) | 1.17 (-0.08,3.44) | 1.93 (-0.14,5.68) | 1.78 (1.73,1.83) |
| Honduras | 34 (-3.18,94.4) | 46.24 (-4.68,121.13) | 0.99 (0.94,1.05) | 2.19 (-0.18,6.3) | 3.13 (-0.27,8.64) | 1.16 (1.08,1.23) |
| Hungary | 32.34 (-3.28,85.33) | 38.07 (-3.94,98.76) | 0.55 (0.54,0.57) | 4.54 (-0.43,12.65) | 5.57 (-0.5,14.9) | 0.68 (0.64,0.71) |
| Iceland | 36.91 (-3.71,100.78) | 44.57 (-4.84,115.96) | 0.65 (0.63,0.68) | 7.12 (-0.66,20.22) | 8.42 (-0.86,23.61) | 0.43 (0.35,0.51) |
| India | 13.32 (-1.03,38.64) | 27.03 (-2.22,77.42) | 2.62 (2.47,2.77) | 0.97 (-0.07,2.79) | 2.25 (-0.17,6.31) | 3.09 (2.97,3.21) |
| Indonesia | 14.26 (-1.11,41.94) | 27.33 (-2.28,76.79) | 2.27 (2.15,2.39) | 1.1 (-0.07,3.25) | 2.16 (-0.15,6.17) | 2.35 (2.23,2.47) |
| Iran (Islamic Republic of) | 26.56 (-2.47,73.86) | 42.62 (-4.88,107.95) | 1.49 (1.46,1.52) | 2.17 (-0.17,5.97) | 3.8 (-0.37,10.25) | 1.73 (1.68,1.79) |
| Iraq | 40.57 (-4.3,106.81) | 47.88 (-5.25,120.15) | 0.57 (0.55,0.59) | 3.07 (-0.29,8.46) | 3.79 (-0.36,10.42) | 0.72 (0.67,0.76) |
| Ireland | 33.9 (-3.45,93.16) | 42.94 (-4.83,110.67) | 0.78 (0.74,0.82) | 5.25 (-0.51,14.78) | 7.34 (-0.74,19.86) | 1.05 (0.99,1.11) |
| Israel | 33.3 (-3.45,92.14) | 40.27 (-4.14,106.86) | 0.59 (0.55,0.63) | 4.78 (-0.43,13.57) | 6.31 (-0.58,17.63) | 0.82 (0.76,0.89) |
| Italy | 28.02 (-2.43,77.17) | 34.92 (-3.51,94.56) | 0.64 (0.61,0.68) | 4.56 (-0.37,12.86) | 6.11 (-0.53,16.8) | 0.95 (0.92,0.98) |
| Jamaica | 39.26 (-3.82,107.76) | 54.31 (-6.27,140.6) | 1.12 (1.05,1.18) | 2.83 (-0.24,8.05) | 4.13 (-0.39,11.7) | 1.32 (1.24,1.4) |
| Japan | 28.41 (-2.45,81.64) | 34.17 (-3.05,94.88) | 0.58 (0.55,0.62) | 2.53 (-0.19,7.21) | 3.3 (-0.25,9.48) | 0.89 (0.82,0.96) |
| Jordan | 40.54 (-4.73,105.51) | 55.23 (-6.84,139.53) | 0.99 (0.96,1.03) | 2.94 (-0.28,8.18) | 4.59 (-0.51,12.24) | 1.44 (1.38,1.49) |
| Kazakhstan | 25.32 (-2.43,67.93) | 31.16 (-3.48,81.39) | 0.65 (0.63,0.66) | 3.87 (-0.34,10.82) | 5.07 (-0.5,13.91) | 0.87 (0.85,0.89) |
| Kenya | 18.08 (-1.41,50.48) | 29 (-2.45,79.34) | 1.54 (1.48,1.59) | 1.94 (-0.14,5.64) | 3.39 (-0.26,9.54) | 1.85 (1.76,1.93) |
| Kiribati | 56.34 (-6.4,145.31) | 70.93 (-9.84,177.69) | 0.68 (0.59,0.76) | 3.6 (-0.37,10.01) | 4.45 (-0.49,12.04) | 0.56 (0.42,0.7) |
| Kuwait | 43.7 (-4.85,112.54) | 60.79 (-8.13,152.72) | 1.11 (1.09,1.13) | 3.67 (-0.38,10.14) | 5.46 (-0.62,14.69) | 1.34 (1.3,1.37) |
| Kyrgyzstan | 23.47 (-2.3,63.05) | 28.81 (-3.17,74.09) | 0.66 (0.64,0.68) | 3.4 (-0.29,9.86) | 4.3 (-0.43,12.06) | 0.72 (0.7,0.75) |
| Lao People's Democratic Republic | 13.68 (-1.01,40.68) | 25.44 (-1.85,73.1) | 2.31 (2.18,2.43) | 0.85 (-0.06,2.55) | 1.62 (-0.11,4.93) | 2.39 (2.27,2.51) |
| Latvia | 32.5 (-3.4,87.24) | 38.46 (-4.2,100.69) | 0.58 (0.56,0.59) | 4.09 (-0.36,11.51) | 5.17 (-0.53,14.95) | 0.79 (0.77,0.81) |
| Lebanon | 35.95 (-3.52,95.07) | 49.81 (-6.2,123.6) | 1.04 (1.02,1.06) | 2.58 (-0.23,7.09) | 4.02 (-0.41,11.14) | 1.34 (1.15,1.53) |
| Lesotho | 27.87 (-2.59,75.04) | 37.76 (-4.03,98.85) | 1.07 (1.03,1.12) | 2.45 (-0.17,7.29) | 3.91 (-0.34,11.06) | 1.58 (1.54,1.62) |
| Liberia | 33.1 (-3,88.74) | 43.87 (-4.43,115.8) | 0.98 (0.94,1.02) | 2.77 (-0.22,8.01) | 3.98 (-0.36,10.94) | 1.37 (1.29,1.45) |
| Libya | 36.42 (-3.85,97.86) | 53.45 (-7.1,135.85) | 1.26 (1.22,1.3) | 2.74 (-0.25,7.86) | 4.32 (-0.49,11.98) | 1.48 (1.44,1.52) |
| Lithuania | 29.68 (-2.82,82.62) | 37.08 (-4.31,96.39) | 0.75 (0.73,0.78) | 3.68 (-0.33,10.65) | 4.9 (-0.48,13.69) | 0.96 (0.9,1.02) |
| Luxembourg | 31.08 (-2.84,85.51) | 39.54 (-3.55,107.05) | 0.77 (0.74,0.79) | 4.91 (-0.42,14.06) | 6.81 (-0.57,18.85) | 1.02 (0.99,1.06) |
| Madagascar | 11.27 (-0.85,34.39) | 18.28 (-1.41,53.6) | 1.57 (1.48,1.66) | 0.99 (-0.06,3.07) | 1.62 (-0.11,5.16) | 1.64 (1.55,1.73) |
| Malawi | 16.22 (-1.28,46.51) | 26.06 (-2.29,71.78) | 1.54 (1.52,1.57) | 1.5 (-0.1,4.54) | 2.52 (-0.19,7.49) | 1.75 (1.71,1.78) |
| Malaysia | 28.63 (-2.65,81.47) | 41.07 (-3.9,109.49) | 1.2 (1.15,1.25) | 1.99 (-0.14,5.88) | 2.98 (-0.24,8.57) | 1.32 (1.26,1.39) |
| Maldives | 23.07 (-1.97,66.61) | 38.96 (-4.16,103.43) | 1.89 (1.78,1.99) | 1.47 (-0.11,4.35) | 2.89 (-0.27,8.19) | 2.35 (2.28,2.43) |
| Mali | 18.04 (-1.46,51.2) | 25.69 (-2.1,71.56) | 1.18 (1.11,1.24) | 1.37 (-0.09,4.06) | 2.07 (-0.16,6.01) | 1.43 (1.36,1.49) |
| Malta | 29.02 (-2.56,81.43) | 40.55 (-4.12,108.36) | 1.06 (0.96,1.16) | 4.36 (-0.36,12.42) | 6.9 (-0.66,19.5) | 1.43 (1.29,1.57) |
| Marshall Islands | 53.72 (-6.28,138.11) | 67.28 (-8.68,167.19) | 0.67 (0.64,0.71) | 3.33 (-0.34,9.06) | 4.16 (-0.42,11.28) | 0.6 (0.52,0.67) |
| Mauritania | 33.36 (-3.17,91.9) | 45.45 (-5.09,119.04) | 0.99 (0.97,1.01) | 2.84 (-0.21,8.04) | 4.22 (-0.41,12.13) | 1.22 (1.18,1.27) |
| Mauritius | 28.78 (-2.55,78.43) | 43.46 (-4.42,114.73) | 1.37 (1.31,1.42) | 2.01 (-0.14,5.67) | 3.13 (-0.29,8.83) | 1.49 (1.41,1.56) |
| Mexico | 43.48 (-4.6,115.07) | 55.58 (-6.55,140.26) | 0.78 (0.76,0.79) | 3.71 (-0.36,10.24) | 4.84 (-0.5,12.89) | 0.7 (0.62,0.78) |
| Micronesia (Federated States of) | 58.03 (-6.53,147.18) | 72.4 (-8.62,178.98) | 0.66 (0.59,0.73) | 3.86 (-0.36,10.91) | 4.76 (-0.52,12.66) | 0.55 (0.42,0.68) |
| Monaco | 37.83 (-3.59,102.85) | 44.61 (-4.85,119.22) | 0.53 (0.51,0.55) | 6.2 (-0.51,17.07) | 7.88 (-0.78,21.93) | 0.75 (0.71,0.8) |
| Mongolia | 21.4 (-2.07,58.14) | 26.02 (-2.69,70.37) | 0.53 (0.45,0.61) | 3.14 (-0.26,9.05) | 3.94 (-0.36,10.87) | 0.8 (0.76,0.84) |
| Montenegro | 31.51 (-3.06,83.7) | 37.89 (-4.19,97.66) | 0.65 (0.63,0.66) | 4.4 (-0.41,12.77) | 5.4 (-0.53,14.96) | 0.75 (0.72,0.77) |
| Morocco | 29.16 (-3.06,80.15) | 42.41 (-4.84,108.6) | 1.25 (1.24,1.27) | 1.87 (-0.16,5.5) | 3.13 (-0.32,8.58) | 1.69 (1.66,1.71) |
| Mozambique | 15.65 (-1.17,46.25) | 26.15 (-2.04,74.1) | 1.72 (1.67,1.77) | 1.38 (-0.09,4.25) | 2.44 (-0.18,7.07) | 1.95 (1.89,2) |
| Myanmar | 17.14 (-1.38,50.2) | 25.81 (-2.3,72.93) | 1.38 (1.31,1.44) | 1.04 (-0.07,3.08) | 1.58 (-0.11,4.61) | 1.45 (1.38,1.52) |
| Namibia | 23.55 (-1.87,66.59) | 36.84 (-3.14,98.58) | 1.43 (1.38,1.48) | 2.28 (-0.15,6.97) | 3.87 (-0.3,10.97) | 1.7 (1.64,1.77) |
| Nauru | 63.83 (-8.26,158.56) | 78.33 (-11.39,194.81) | 0.56 (0.52,0.61) | 4.52 (-0.48,12.44) | 5.3 (-0.69,14.13) | 0.38 (0.32,0.44) |
| Nepal | 15.9 (-1.14,47.54) | 32.8 (-2.91,90) | 2.58 (2.45,2.71) | 1.09 (-0.08,3.33) | 2.69 (-0.21,8.09) | 3.25 (3.09,3.4) |
| Netherlands | 32.08 (-3.16,89.77) | 41.58 (-4.33,110.1) | 0.86 (0.83,0.89) | 4.9 (-0.47,13.73) | 6.64 (-0.59,18.55) | 1 (0.97,1.04) |
| New Zealand | 39 (-3.73,104.39) | 50.57 (-5.63,131.93) | 0.88 (0.85,0.91) | 5.68 (-0.49,15.69) | 8.27 (-0.83,22.73) | 1.25 (1.19,1.31) |
| Nicaragua | 41.03 (-4.06,111.08) | 53.33 (-5.92,139.91) | 0.85 (0.81,0.89) | 2.74 (-0.2,7.58) | 3.72 (-0.37,10.45) | 0.95 (0.9,1.01) |
| Niger | 19.3 (-1.52,54.66) | 24.65 (-2.22,71.96) | 0.73 (0.7,0.75) | 1.45 (-0.09,4.3) | 1.85 (-0.14,5.52) | 0.79 (0.76,0.81) |
| Nigeria | 20.76 (-1.75,59.06) | 33.52 (-3.21,88.76) | 1.55 (1.39,1.72) | 2.03 (-0.15,5.71) | 3.12 (-0.26,8.68) | 1.17 (1.04,1.31) |
| Niue | 60.93 (-6.8,155.45) | 77.9 (-9.55,191) | 0.75 (0.69,0.81) | 4.21 (-0.4,11.7) | 5.59 (-0.6,14.94) | 0.85 (0.75,0.96) |
| North Macedonia | 29.61 (-3,78.82) | 35.62 (-3.85,92.65) | 0.64 (0.63,0.66) | 3.94 (-0.35,10.98) | 4.98 (-0.47,14.25) | 0.81 (0.79,0.83) |
| Northern Mariana Islands | 63.21 (-6.95,162.4) | 77.66 (-9.66,196.58) | 0.64 (0.55,0.73) | 4.95 (-0.52,13.49) | 5.7 (-0.67,14.61) | 0.27 (0.15,0.39) |
| Norway | 28.7 (-2.63,79.89) | 35.65 (-3.43,95.5) | 0.71 (0.69,0.73) | 4.95 (-0.4,13.85) | 6.68 (-0.59,18.54) | 1 (0.98,1.01) |
| Oman | 30.6 (-3.01,82.87) | 53.81 (-6.56,135.57) | 1.96 (1.91,2.01) | 2.17 (-0.2,6.27) | 4.67 (-0.5,13.15) | 2.65 (2.6,2.71) |
| Pakistan | 18.09 (-1.49,50.75) | 30.84 (-2.88,86.69) | 2.01 (1.91,2.11) | 1.84 (-0.14,5.17) | 3.55 (-0.3,9.95) | 2.46 (2.36,2.57) |
| Palau | 60.86 (-6.66,156.95) | 74.26 (-9.16,186.75) | 0.59 (0.54,0.63) | 4.33 (-0.42,11.67) | 5.56 (-0.63,15.12) | 0.62 (0.48,0.77) |
| Palestine | 39.49 (-4.15,103.98) | 50.1 (-6.22,124.92) | 0.74 (0.7,0.78) | 2.7 (-0.26,7.47) | 3.79 (-0.4,10.13) | 0.99 (0.93,1.05) |
| Panama | 29.2 (-2.58,81.98) | 46.93 (-4.33,125.91) | 1.56 (1.48,1.63) | 1.83 (-0.14,5.36) | 3.34 (-0.27,9.79) | 1.94 (1.87,2.01) |
| Papua New Guinea | 32.39 (-2.86,90.49) | 43.62 (-4.46,122.39) | 0.9 (0.84,0.97) | 1.89 (-0.14,5.51) | 2.49 (-0.21,7.2) | 0.8 (0.71,0.89) |
| Paraguay | 38.5 (-4.11,102.49) | 50.16 (-5.42,133.89) | 0.88 (0.87,0.9) | 2.8 (-0.23,8.08) | 3.76 (-0.34,10.26) | 1 (0.97,1.02) |
| Peru | 38.81 (-4.01,104.83) | 53.49 (-6.15,138.1) | 1.08 (1.06,1.09) | 2.7 (-0.22,7.62) | 3.92 (-0.37,10.86) | 1.28 (1.26,1.31) |
| Philippines | 16.69 (-1.3,47.5) | 25.8 (-2.14,71.84) | 1.38 (1.3,1.46) | 1.51 (-0.1,4.32) | 2.31 (-0.16,6.57) | 1.33 (1.27,1.38) |
| Poland | 25.76 (-2.62,69.71) | 31.83 (-3.35,84.59) | 0.7 (0.68,0.72) | 3.93 (-0.34,11.02) | 5.23 (-0.5,14.27) | 0.94 (0.9,0.97) |
| Portugal | 31.14 (-2.95,87.53) | 41.33 (-4.47,109.61) | 0.84 (0.77,0.91) | 4.5 (-0.4,12.78) | 6.64 (-0.62,18.73) | 1.03 (0.91,1.14) |
| Puerto Rico | 51.73 (-5.5,134.45) | 64.97 (-8.01,167.56) | 0.77 (0.73,0.82) | 4.4 (-0.41,12.07) | 5.71 (-0.59,15.63) | 0.95 (0.89,1) |
| Qatar | 43.37 (-5.07,112.96) | 58.71 (-7.88,148.3) | 0.94 (0.91,0.96) | 3.89 (-0.4,11.03) | 5.8 (-0.67,15.66) | 1.24 (1.17,1.31) |
| Republic of Korea | 28.63 (-2.65,83.76) | 42.54 (-4.21,121.5) | 1.43 (1.31,1.54) | 2.17 (-0.17,6.28) | 3.36 (-0.29,9.34) | 1.42 (1.37,1.47) |
| Republic of Moldova | 30.13 (-2.98,81.36) | 39.53 (-4.47,100.33) | 0.97 (0.94,1) | 3.55 (-0.33,10.21) | 5.18 (-0.51,14.41) | 1.34 (1.3,1.39) |
| Romania | 27.56 (-2.41,75.05) | 34.52 (-3.12,94.13) | 0.75 (0.72,0.78) | 3.63 (-0.28,10.36) | 4.97 (-0.4,13.55) | 1.05 (1,1.09) |
| Russian Federation | 28 (-2.73,75.31) | 36.63 (-4.09,94.34) | 0.92 (0.9,0.94) | 3.92 (-0.32,10.89) | 5.52 (-0.51,14.93) | 1.18 (1.16,1.2) |
| Rwanda | 14.11 (-1.14,40.95) | 21.47 (-1.87,62.21) | 1.33 (1.24,1.42) | 1.25 (-0.09,3.79) | 2.03 (-0.15,6.13) | 1.57 (1.46,1.67) |
| Saint Kitts and Nevis | 40.87 (-3.82,110.22) | 55.72 (-6.45,141.38) | 1.01 (0.99,1.04) | 3.16 (-0.26,9.15) | 4.52 (-0.43,12.53) | 1.2 (1.16,1.24) |
| Saint Lucia | 38.96 (-4.07,104.38) | 53.31 (-5.81,140.44) | 1.01 (0.98,1.05) | 2.83 (-0.23,8.05) | 4.09 (-0.38,11.28) | 1.19 (1.14,1.24) |
| Saint Vincent and the Grenadines | 31.8 (-2.95,87.85) | 46 (-4.39,123.08) | 1.25 (1.23,1.27) | 2.24 (-0.18,6.57) | 3.52 (-0.32,9.86) | 1.54 (1.5,1.59) |
| Samoa | 65.67 (-8.09,163.62) | 76.06 (-10.37,189.04) | 0.39 (0.34,0.45) | 4.55 (-0.48,12.23) | 5.28 (-0.63,13.99) | 0.31 (0.21,0.41) |
| San Marino | 35.32 (-3.12,96.13) | 43.35 (-4.71,115.53) | 0.67 (0.64,0.7) | 5.71 (-0.49,16.46) | 7.39 (-0.7,20.14) | 0.81 (0.77,0.86) |
| Sao Tome and Principe | 29.33 (-2.96,82.69) | 41.38 (-4.1,110.08) | 1.09 (1.08,1.1) | 2.36 (-0.18,6.98) | 3.69 (-0.32,10.4) | 1.46 (1.44,1.48) |
| Saudi Arabia | 37.11 (-3.87,99.28) | 57 (-7.03,140.84) | 1.4 (1.36,1.44) | 2.81 (-0.26,8.01) | 5.09 (-0.56,13.69) | 1.92 (1.86,1.98) |
| Senegal | 27.32 (-2.35,76.25) | 34.96 (-3.27,94.19) | 0.73 (0.71,0.75) | 2.18 (-0.17,6.43) | 2.92 (-0.22,8.62) | 0.87 (0.83,0.91) |
| Serbia | 28.27 (-2.74,77.34) | 36.61 (-3.84,94.99) | 0.88 (0.86,0.9) | 3.76 (-0.31,10.41) | 5.22 (-0.48,14.29) | 1.13 (1.11,1.16) |
| Seychelles | 36.24 (-3.76,96.12) | 50.96 (-6.22,132.76) | 1.12 (1.08,1.17) | 2.57 (-0.24,7.16) | 3.79 (-0.41,10.73) | 1.24 (1.18,1.29) |
| Sierra Leone | 20.39 (-1.67,57.11) | 29.51 (-2.61,82.9) | 1.14 (1.07,1.22) | 1.57 (-0.1,4.75) | 2.44 (-0.19,7.3) | 1.38 (1.26,1.5) |
| Singapore | 31.51 (-2.67,92.04) | 51.63 (-4.57,144.3) | 1.49 (1.38,1.6) | 2.43 (-0.17,7.09) | 4.49 (-0.34,12.69) | 1.95 (1.81,2.09) |
| Slovakia | 31.05 (-3.21,82.13) | 35.94 (-3.75,93.96) | 0.45 (0.43,0.47) | 4.19 (-0.38,11.89) | 5.18 (-0.5,14.61) | 0.61 (0.58,0.65) |
| Slovenia | 29.88 (-2.97,80.59) | 36.01 (-3.91,95.77) | 0.65 (0.63,0.67) | 4.1 (-0.32,11.33) | 5.27 (-0.49,14.28) | 0.85 (0.81,0.9) |
| Solomon Islands | 43 (-3.88,115.93) | 56.33 (-6.01,144.55) | 0.8 (0.73,0.86) | 2.57 (-0.2,7.49) | 3.37 (-0.31,9.35) | 0.75 (0.63,0.86) |
| Somalia | 17.63 (-1.31,51.75) | 24.09 (-2.07,66.51) | 1.09 (1.06,1.13) | 1.51 (-0.1,4.5) | 2.1 (-0.16,6.13) | 1.15 (1.11,1.2) |
| South Africa | 34.86 (-3.36,92.76) | 45.27 (-5.15,114.35) | 0.83 (0.82,0.84) | 4.19 (-0.34,11.61) | 5.79 (-0.55,15.94) | 1.05 (1.03,1.07) |
| South Sudan | 11.79 (-0.87,35.3) | 16.45 (-1.34,48.86) | 1.05 (0.99,1.1) | 1.07 (-0.07,3.34) | 1.55 (-0.11,4.65) | 1.17 (1.11,1.24) |
| Spain | 34.24 (-3.28,94.17) | 42.71 (-4.36,114.82) | 0.75 (0.71,0.78) | 5.5 (-0.49,15.55) | 7.58 (-0.77,21.19) | 0.9 (0.77,1.03) |
| Sri Lanka | 19.78 (-1.55,58.54) | 30.8 (-2.59,87.9) | 1.44 (1.39,1.48) | 1.29 (-0.09,3.83) | 2.04 (-0.13,6.02) | 1.5 (1.46,1.53) |
| Sudan | 28.21 (-2.7,76.36) | 44.65 (-4.9,115.01) | 1.5 (1.47,1.54) | 1.74 (-0.14,4.9) | 3.27 (-0.32,9.33) | 2.09 (2.04,2.14) |
| Suriname | 32.7 (-3.09,92.1) | 46.39 (-4.98,123.8) | 1.13 (1.11,1.14) | 2.44 (-0.18,7.18) | 3.61 (-0.33,10.01) | 1.33 (1.31,1.35) |
| Sweden | 23.45 (-2.45,64.62) | 30.64 (-3.13,83.6) | 0.9 (0.86,0.94) | 4.49 (-0.4,12.5) | 6.94 (-0.62,19.63) | 1.37 (1.26,1.49) |
| Switzerland | 28.87 (-2.67,79.96) | 34.71 (-3.46,96.49) | 0.62 (0.61,0.64) | 4.46 (-0.35,12.58) | 5.67 (-0.5,16.19) | 0.8 (0.78,0.82) |
| Syrian Arab Republic | 36.19 (-3.76,95.89) | 52.37 (-7,131.86) | 1.21 (1.18,1.24) | 2.52 (-0.23,6.97) | 3.99 (-0.47,10.71) | 1.43 (1.36,1.49) |
| Taiwan (Province of China) | 34.77 (-2.96,101.98) | 63.76 (-6.72,175.81) | 2.06 (2,2.11) | 1.46 (-0.11,4.34) | 2.63 (-0.23,7.41) | 2.05 (1.97,2.12) |
| Tajikistan | 22.24 (-2.3,60.72) | 26.37 (-3.03,70.35) | 0.57 (0.56,0.59) | 3.09 (-0.24,8.82) | 3.68 (-0.35,10.14) | 0.58 (0.56,0.61) |
| Thailand | 22.44 (-1.87,64.47) | 42.49 (-4.48,117.25) | 2.13 (2.05,2.21) | 1.37 (-0.08,4.13) | 2.72 (-0.24,7.76) | 2.32 (2.24,2.4) |
| Timor-Leste | 8.42 (-0.69,26.23) | 15.85 (-1.1,46.1) | 2.13 (2.07,2.19) | 0.54 (-0.04,1.67) | 0.99 (-0.06,2.99) | 2.08 (2,2.16) |
| Togo | 22.7 (-2.01,63.84) | 32.9 (-2.73,89.64) | 1.13 (1.09,1.17) | 1.73 (-0.12,5.09) | 2.71 (-0.19,7.79) | 1.39 (1.34,1.45) |
| Tokelau | 57.01 (-6.46,147.2) | 73.65 (-8.96,180.85) | 0.8 (0.76,0.84) | 3.63 (-0.38,9.91) | 5.14 (-0.59,13.94) | 1.04 (0.95,1.13) |
| Tonga | 67.76 (-8.03,172.12) | 81.4 (-11.14,199.68) | 0.48 (0.4,0.56) | 4.47 (-0.46,12.12) | 5.54 (-0.66,14.25) | 0.47 (0.3,0.64) |
| Trinidad and Tobago | 42.48 (-4.06,113.23) | 55.48 (-6.84,143.71) | 0.92 (0.9,0.94) | 3.31 (-0.27,9.3) | 4.41 (-0.46,12.43) | 1.07 (1.02,1.11) |
| Türkiye | 38.55 (-4.15,102.62) | 54.03 (-6.64,138.65) | 1.08 (1.06,1.1) | 2.64 (-0.26,7.56) | 4.15 (-0.45,11.25) | 1.43 (1.4,1.46) |
| Tunisia | 31.23 (-2.91,85.91) | 46.58 (-5.63,119.87) | 1.33 (1.31,1.35) | 2.13 (-0.18,6.04) | 3.53 (-0.37,9.66) | 1.66 (1.65,1.68) |
| Turkmenistan | 23.39 (-2.29,62.8) | 27.79 (-2.99,74.67) | 0.58 (0.57,0.59) | 3.48 (-0.3,9.91) | 4.48 (-0.39,12.7) | 0.82 (0.8,0.84) |
| Tuvalu | 53.81 (-5.75,139.24) | 69.82 (-8.67,174.48) | 0.8 (0.75,0.84) | 3.39 (-0.33,9.41) | 4.74 (-0.52,12.82) | 0.98 (0.9,1.07) |
| Uganda | 16.15 (-1.23,47.7) | 26.02 (-2.31,72.92) | 1.62 (1.6,1.64) | 1.43 (-0.1,4.21) | 2.42 (-0.18,7.22) | 1.83 (1.79,1.87) |
| Ukraine | 28.83 (-2.62,77.69) | 35.18 (-3.82,93.46) | 0.69 (0.67,0.71) | 4.11 (-0.29,11.74) | 5.15 (-0.49,14.72) | 0.81 (0.78,0.83) |
| United Arab Emirates | 36.46 (-3.44,98.69) | 56.8 (-7.12,141.26) | 1.48 (1.44,1.52) | 3.03 (-0.29,8.6) | 5.33 (-0.65,14.12) | 1.93 (1.89,1.97) |
| United Kingdom | 36.44 (-3.64,98.8) | 46 (-4.95,119.96) | 0.79 (0.71,0.87) | 6.19 (-0.56,17.05) | 8.32 (-0.81,22.48) | 0.97 (0.92,1.03) |
| United Republic of Tanzania | 20.48 (-1.74,59.92) | 31.53 (-2.62,87.36) | 1.42 (1.39,1.44) | 1.82 (-0.12,5.35) | 3.07 (-0.22,8.82) | 1.72 (1.69,1.74) |
| United States of America | 51.84 (-5.4,138.39) | 62.89 (-7.47,160.09) | 0.67 (0.52,0.82) | 8.32 (-0.79,22.82) | 10.95 (-1.15,29.5) | 1.3 (1.17,1.44) |
| United States Virgin Islands | 51.49 (-5.64,133.44) | 61.98 (-6.97,158.03) | 0.62 (0.59,0.65) | 4.34 (-0.43,12.22) | 5.34 (-0.52,14.04) | 0.69 (0.65,0.72) |
| Uruguay | 39.12 (-3.99,107.59) | 51.45 (-5.64,134.12) | 0.88 (0.84,0.93) | 4.74 (-0.43,13.48) | 7.2 (-0.68,19.92) | 1.43 (1.33,1.52) |
| Uzbekistan | 23.02 (-2.42,63.03) | 29.25 (-3.16,76.85) | 0.82 (0.81,0.83) | 3.32 (-0.29,9.81) | 4.5 (-0.41,12.65) | 1.05 (1.02,1.07) |
| Vanuatu | 41.32 (-3.91,110.21) | 55.18 (-5.85,142.91) | 0.91 (0.86,0.96) | 2.61 (-0.22,7.46) | 3.38 (-0.35,9.44) | 0.83 (0.78,0.88) |
| Venezuela (Bolivarian Republic of) | 43.22 (-4.42,115.38) | 55.26 (-6.18,141) | 0.81 (0.8,0.82) | 3.07 (-0.26,8.6) | 4.01 (-0.38,11.15) | 0.9 (0.87,0.92) |
| Viet Nam | 8.14 (-0.62,24.68) | 17.84 (-1.41,53.41) | 2.98 (2.79,3.16) | 0.5 (-0.04,1.52) | 1.09 (-0.07,3.44) | 3.01 (2.81,3.21) |
| Yemen | 20.23 (-1.53,57.58) | 32.29 (-3.15,88.59) | 1.59 (1.57,1.62) | 1.21 (-0.08,3.73) | 2.12 (-0.18,5.94) | 1.94 (1.9,1.99) |
| Zambia | 18.11 (-1.51,51.24) | 30.45 (-2.71,84.78) | 1.68 (1.66,1.7) | 1.7 (-0.13,5.01) | 3.03 (-0.22,9.01) | 1.84 (1.79,1.9) |
| Zimbabwe | 22.52 (-1.9,63.6) | 32.71 (-2.84,87.47) | 1.1 (1.01,1.19) | 2.12 (-0.15,6.36) | 3.17 (-0.23,8.98) | 1.11 (1.02,1.21) |
