# Supplementary material for: Global, regional, and national burden of early-onset OA attributable to high BMI: 1990–2021 estimates and 2036 projections from the global burden of disease study
Source: PLoS One. 2025 Jul 16;20(7):e0328414. doi: 10.1371/journal.pone.0328414 (PMC12266449; doi:10.1371/journal.pone.0328414)
Supplement: S4 Table — (DOCX) [file pone.0328414.s011.docx]

| Table S4. **Joinpoint regression analysis was conducted on the ASDR for both sexes.** | | | | | | | |
| --- | --- | --- | --- | --- | --- | --- | --- |
| **Sex name** | **Knee OA** | | |  | **Hip OA** | | |
|  | **Range** | **APC (95% CI)** | ***P*** | **Sex name** | **Range** | **APC (95% CI)** | ***P*** |
| Both | 1990-1999 | 1.260 (1.229 - 1.290) | < 0.001 | Both | 1990-1995 | 0.515 (0.268 - 0.762) | < 0.001 |
| Both | 1999-2005 | 1.630 (1.555 - 1.705) | < 0.001 | Both | 1995-2000 | 1.885 (1.529 - 2.242) | < 0.001 |
| Both | 2005-2009 | 2.447 (2.281 - 2.615) | < 0.001 | Both | 2000-2005 | 0.891 (0.537 - 1.247) | < 0.001 |
| Both | 2009-2015 | 1.667 (1.594 - 1.741) | < 0.001 | Both | 2005-2010 | 1.557 (1.200 - 1.915) | < 0.001 |
| Both | 2015-2019 | 0.118 (-0.043 - 0.280) | 0.139 | Both | 2010-2015 | 0.778 (0.425 - 1.131) | < 0.001 |
| Both | 2019-2021 | 0.946 (0.622 - 1.271) | < 0.001 | Both | 2015-2021 | 1.196 (1.006 - 1.385) | < 0.001 |
| Female | 1990-1996 | 1.197 (1.151 - 1.243) | < 0.001 | Female | 1990-1995 | 0.557 (0.331 - 0.783) | < 0.001 |
| Female | 1996-2005 | 1.438 (1.409 - 1.468) | < 0.001 | Female | 1995-2000 | 1.727 (1.403 - 2.052) | < 0.001 |
| Female | 2005-2009 | 2.625 (2.488 - 2.762) | < 0.001 | Female | 2000-2005 | 0.843 (0.520 - 1.167) | < 0.001 |
| Female | 2009-2015 | 1.706 (1.646 - 1.766) | < 0.001 | Female | 2005-2010 | 1.713 (1.387 - 2.040) | < 0.001 |
| Female | 2015-2019 | 0.058 (-0.073 - 0.190) | 0.359 | Female | 2010-2021 | 1.006 (0.938 - 1.075) | < 0.001 |
| Female | 2019-2021 | 0.725 (0.462 - 0.990) | < 0.001 | Male | 1990-1995 | 0.463 (0.263 - 0.663) | < 0.001 |
| Male | 1990-2000 | 1.263 (1.240 - 1.286) | < 0.001 | Male | 1995-1999 | 2.104 (1.652 - 2.559) | < 0.001 |
| Male | 20002005 | 1.857 (1.765 - 1.950) | < 0.001 | Male | 1999-2011 | 1.180 (1.119 - 1.241) | < 0.001 |
| Male | 2005-2009 | 2.154 (2.008 - 2.299) | < 0.001 | Male | 2011-2015 | 0.532 (0.083 - 0.982) | 0.023 |
| Male | 2009-2015 | 1.567 (1.503 - 1.632) | < 0.001 | Male | 2015-2019 | 1.611 (1.158 - 2.066) | < 0.001 |
| Male | 2015-2019 | 0.216 (0.074 - 0.358) | 0.005 | Male | 2019-2021 | 0.342 (-0.550 - 1.241) | 0.428 |
| Male | 2019-2021 | 1.300 (1.015 - 1.586) | < 0.001 |  |  |  |  |
|  | | | | | | | |
| **Sex name** | **Range** | **AAPC (95% CI)** | ***P*** | **Sex name** | **Range** | **AAPC (95% CI)** | ***P*** |
| Both | 1990-2021 | 1.394 (1.355 - 1.433) | < 0.001 | Both | 1990-2021 | 1.138 (1.021 - 1.254) | < 0.001 |
| Female | 1990-2021 | 1.370 (1.339 - 1.402) | < 0.001 | Female | 1990-2021 | 1.137 (1.043 - 1.231) | < 0.001 |
| Male | 1990-2021 | 1.399 (1.363 - 1.434) | < 0.001 | Male | 1990-2021 | 1.100 (0.987 - 1.213) | < 0.001 |
