# Supplementary material for: Global, regional, and national burden of early-onset OA attributable to high BMI: 1990–2021 estimates and 2036 projections from the global burden of disease study
Source: PLoS One. 2025 Jul 16;20(7):e0328414. doi: 10.1371/journal.pone.0328414 (PMC12266449; doi:10.1371/journal.pone.0328414)
Supplement: S5 Table — (DOCX) [file pone.0328414.s012.docx]

| Table S5. The effects of age, period, and birth cohort on the relative risk of DALYs for early-onset OA attributable to high BMI. | | | | | | | | | | | | |
| --- | --- | --- | --- | --- | --- | --- | --- | --- | --- | --- | --- | --- |
| **Factor** | **Knee OA** | | | | | | **Hip OA** | | | | | |
|  | **Both** |  | **Male** |  | **Female** |  | **Both** |  | **Male** |  | **Female** |  |
|  | **Rate** | ***P*** | **Rate** | ***P*** | **Rate** | ***P*** | **Rate** | ***P*** | **Rate** | ***P*** | **Rate** | ***P*** |
|  | **(95% CI)** |  | **(95% CI)** |  | **(95% CI)** |  | **(95% CI)** |  | **(95% CI)** |  | **(95% CI)** |  |
| **Age (years)** | | | | | | | | | | | | |
| **30-34** | 0.737 (0.704,0.772) | < 0.001 | 0.612 (0.589,0.636) | < 0.001 | 0.865 (0.816,0.917) | < 0.001 | 0.649 (0.637,0.66) | < 0.001 | 0.676 (0.66,0.694) | < 0.001 | 0.62 (0.604,0.637) | < 0.001 |
| **35-39** | 8.169 (8.027,8.313) | < 0.001 | 6.705 (6.609,6.801) | < 0.001 | 9.667 (9.457,9.883) | < 0.001 | 1.311 (1.293,1.329) | < 0.001 | 1.368 (1.342,1.394) | < 0.001 | 1.253 (1.229,1.278) | < 0.001 |
| **40-44** | 26.901 (26.586,27.219) | < 0.001 | 21.548 (21.34,21.758) | < 0.001 | 32.383 (31.911,32.862) | < 0.001 | 2.526 (2.498,2.554) | < 0.001 | 2.619 (2.579,2.66) | < 0.001 | 2.431 (2.392,2.47) | < 0.001 |
| **45-49** | 60.75 (60.18,61.326) | < 0.001 | 47.104 (46.734,47.477) | < 0.001 | 74.604 (73.736,75.482) | < 0.001 | 4.74 (4.695,4.785) | < 0.001 | 4.836 (4.772,4.901) | < 0.001 | 4.641 (4.577,4.705) | < 0.001 |
| **50-54** | 107.455 (106.549,108.369) | < 0.001 | 82.448 (81.862,83.037) | < 0.001 | 132.355 (130.979,133.745) | < 0.001 | 8.232 (8.16,8.305) | < 0.001 | 8.339 (8.237,8.442) | < 0.001 | 8.124 (8.023,8.227) | < 0.001 |
|  |  |  |  |  |  |  |  |  |  |  |  |  |
|  | **RRs** | **P** | **RRs** | **P** | **RRs** | **P** | **RRs** | **P** | **RRs** | **P** | **RRs** | ***P*** |
|  | **(95% CI)** |  | **(95% CI)** |  | **(95% CI)** |  | **(95% CI)** |  | **(95% CI)** |  | **(95% CI)** |  |
| **Period** | | | | | | | | | | | | |
| **1994** | 1 (1,1) | < 0.001 | 1 (1,1) | < 0.001 | 1 (1,1) | < 0.001 | 1 (1,1) | < 0.001 | 1 (1,1) | < 0.001 | 1 (1,1) | < 0.001 |
| **1999** | 1.07 (1.053,1.088) | < 0.001 | 1.068 (1.054,1.083) | < 0.001 | 1.072 (1.05,1.094) | < 0.001 | 1.08 (1.064,1.097) | < 0.001 | 1.08 (1.058,1.103) | < 0.001 | 1.081 (1.058,1.105) | < 0.001 |
| **2004** | 1.164 (1.143,1.185) | < 0.001 | 1.167 (1.15,1.185) | < 0.001 | 1.161 (1.135,1.187) | < 0.001 | 1.158 (1.14,1.176) | < 0.001 | 1.157 (1.133,1.182) | < 0.001 | 1.159 (1.133,1.185) | < 0.001 |
| **2009** | 1.299 (1.274,1.325) | < 0.001 | 1.294 (1.274,1.315) | < 0.001 | 1.3 (1.269,1.332) | < 0.001 | 1.247 (1.228,1.266) | < 0.001 | 1.237 (1.212,1.264) | < 0.001 | 1.259 (1.231,1.287) | < 0.001 |
| **2014** | 1.403 (1.375,1.432) | < 0.001 | 1.395 (1.373,1.419) | < 0.001 | 1.404 (1.369,1.441) | < 0.001 | 1.317 (1.298,1.336) | < 0.001 | 1.299 (1.273,1.325) | < 0.001 | 1.337 (1.31,1.366) | < 0.001 |
| **2019** | 1.439 (1.409,1.469) | < 0.001 | 1.438 (1.414,1.462) | < 0.001 | 1.434 (1.397,1.472) | < 0.001 | 1.394 (1.375,1.413) | < 0.001 | 1.382 (1.356,1.408) | < 0.001 | 1.408 (1.381,1.436) | < 0.001 |
| **Birth cohort** | | | | | | | | | | | | |
| 1938—1946 | 0.746 (0.731,0.761) | < 0.001 | 0.747 (0.735,0.76) | < 0.001 | 0.749 (0.731,0.768) | < 0.001 | 0.789 (0.773,0.805) | < 0.001 | 0.782 (0.76,0.804) | < 0.001 | 0.796 (0.773,0.819) | < 0.001 |
| 1943—1951 | 0.805 (0.793,0.816) | < 0.001 | 0.805 (0.796,0.815) | < 0.001 | 0.808 (0.794,0.823) | < 0.001 | 0.852 (0.839,0.865) | < 0.001 | 0.847 (0.83,0.865) | < 0.001 | 0.857 (0.839,0.876) | < 0.001 |
| 1948—1956 | 0.865 (0.854,0.876) | < 0.001 | 0.866 (0.857,0.875) | < 0.001 | 0.866 (0.852,0.88) | < 0.001 | 0.89 (0.878,0.901) | < 0.001 | 0.889 (0.873,0.905) | < 0.001 | 0.89 (0.874,0.907) | < 0.001 |
| 1953—1961 | 0.921 (0.911,0.932) | < 0.001 | 0.926 (0.917,0.935) | < 0.001 | 0.919 (0.906,0.933) | < 0.001 | 0.952 (0.941,0.963) | < 0.001 | 0.952 (0.936,0.967) | < 0.001 | 0.952 (0.936,0.968) | < 0.001 |
| 1958—1966 | 1 (1,1) | < 0.001 | 1 (1,1) | < 0.001 | 1 (1,1) | < 0.001 | 1 (1,1) | < 0.001 | 1 (1,1) | < 0.001 | 1 (1,1) | < 0.001 |
| 1963—1971 | 1.107 (1.095,1.119) | < 0.001 | 1.097 (1.087,1.107) | < 0.001 | 1.113 (1.098,1.128) | < 0.001 | 1.037 (1.026,1.048) | < 0.001 | 1.032 (1.017,1.048) | < 0.001 | 1.042 (1.026,1.058) | < 0.001 |
| 1968—1976 | 1.189 (1.173,1.206) | < 0.001 | 1.187 (1.173,1.2) | < 0.001 | 1.19 (1.17,1.211) | < 0.001 | 1.128 (1.114,1.143) | < 0.001 | 1.117 (1.097,1.137) | < 0.001 | 1.141 (1.12,1.162) | < 0.001 |
| 1973—1981 | 1.269 (1.244,1.293) | < 0.001 | 1.269 (1.249,1.289) | < 0.001 | 1.267 (1.237,1.298) | < 0.001 | 1.248 (1.228,1.268) | < 0.001 | 1.223 (1.196,1.251) | < 0.001 | 1.275 (1.246,1.305) | < 0.001 |
| 1978—1986 | 1.369 (1.325,1.414) | < 0.001 | 1.373 (1.337,1.41) | < 0.001 | 1.364 (1.309,1.422) | < 0.001 | 1.367 (1.338,1.397) | < 0.001 | 1.338 (1.298,1.379) | < 0.001 | 1.401 (1.358,1.445) | < 0.001 |
| 1983—1991 | 1.508 (1.372,1.658) | < 0.001 | 1.505 (1.394,1.625) | < 0.001 | 1.51 (1.34,1.702) | < 0.001 | 1.487 (1.438,1.537) | < 0.001 | 1.454 (1.388,1.523) | < 0.001 | 1.524 (1.453,1.599) | < 0.001 |
