# Supplementary material for: Global, regional, and national burden of early-onset OA attributable to high BMI: 1990–2021 estimates and 2036 projections from the global burden of disease study
Source: PLoS One. 2025 Jul 16;20(7):e0328414. doi: 10.1371/journal.pone.0328414 (PMC12266449; doi:10.1371/journal.pone.0328414)
Supplement: S6 Table — (DOCX) [file pone.0328414.s013.docx]

| Table S6. **Decomposition analysis of changes in the DALYs of early-onset OA attributable to high BMI, stratified by gender from 1990 to 2021.** | | | | | | | | |
| --- | --- | --- | --- | --- | --- | --- | --- | --- |
|  | **sex_name** | **Overall difference** | **Aging** | **Population** | **Epidemiological change** | **Aging percent (%)** | **Population percent (%)** | **Epidemiological change percent (%)** |
| **Knee osteoarthritis** | Both | 735167.54 | 80384.304 | 371187.249 | 283595.985 | 10.93 | 50.49 | 38.58 |
|  | Male | 285293.5 | 29853.018 | 143750.594 | 111689.893 | 10.46 | 50.39 | 39.15 |
|  | Female | 449874.03 | 51206.154 | 228775.613 | 169892.267 | 11.38 | 50.85 | 37.76 |
| **Hip osteoarthritis** | Both | 60359.4 | 5353.327 | 34014.498 | 20991.577 | 8.87 | 56.35 | 34.78 |
|  | Male | 30433.63 | 2608.938 | 17169.159 | 10655.535 | 8.57 | 56.42 | 35.01 |
|  | Female | 29925.77 | 2741.97 | 16835.852 | 10347.949 | 9.16 | 56.26 | 34.58 |
