# Supplementary material for: Global, regional, and national burden of early-onset OA attributable to high BMI: 1990–2021 estimates and 2036 projections from the global burden of disease study
Source: PLoS One. 2025 Jul 16;20(7):e0328414. doi: 10.1371/journal.pone.0328414 (PMC12266449; doi:10.1371/journal.pone.0328414)
Supplement: S7 Table — (DOCX) [file pone.0328414.s014.docx]

| Table S7. The projected ASDR of early-onset osteoarthritis attributable to high BMI over the next 15 years. | | | | | | |
| --- | --- | --- | --- | --- | --- | --- |
| Year | Knee OA (95% CI) | | | Hip OA (95% CI) | | |
|  | Both | Female | Male | Both | Female | Male |
| 2022 | 41.205 (40.98,41.429) | 49.836 (49.606,50.066) | 32.321 (32.112,32.53) | 3.672 (3.647,3.696) | 3.601 (3.579,3.623) | 3.738 (3.711,3.765) |
| 2023 | 41.263 (40.706,41.82) | 49.399 (48.712,50.087) | 32.62 (32.201,33.039) | 3.706 (3.654,3.758) | 3.643 (3.595,3.691) | 3.777 (3.716,3.838) |
| 2024 | 41.53 (40.543,42.518) | 49.097 (47.844,50.35) | 32.93 (32.296,33.565) | 3.74 (3.671,3.809) | 3.681 (3.611,3.752) | 3.812 (3.727,3.897) |
| 2025 | 41.888 (40.519,43.256) | 49.103 (47.31,50.896) | 33.249 (32.403,34.095) | 3.774 (3.691,3.856) | 3.716 (3.628,3.804) | 3.846 (3.742,3.951) |
| 2026 | 42.284 (40.586,43.981) | 49.273 (47.009,51.537) | 33.573 (32.524,34.623) | 3.808 (3.713,3.902) | 3.749 (3.648,3.851) | 3.881 (3.76,4.002) |
| 2027 | 42.696 (40.712,44.681) | 49.354 (46.646,52.062) | 33.902 (32.659,35.145) | 3.842 (3.737,3.947) | 3.782 (3.67,3.894) | 3.915 (3.78,4.05) |
| 2028 | 43.116 (40.876,45.356) | 49.238 (46.048,52.428) | 34.234 (32.807,35.661) | 3.876 (3.761,3.99) | 3.815 (3.694,3.936) | 3.95 (3.802,4.097) |
| 2029 | 43.539 (41.068,46.01) | 49.023 (45.28,52.766) | 34.568 (32.969,36.168) | 3.91 (3.786,4.033) | 3.849 (3.719,3.978) | 3.984 (3.824,4.144) |
| 2030 | 43.963 (41.28,46.647) | 48.872 (44.522,53.222) | 34.905 (33.141,36.668) | 3.944 (3.812,4.075) | 3.883 (3.745,4.02) | 4.018 (3.847,4.189) |
| 2031 | 44.388 (41.508,47.268) | 48.853 (43.884,53.821) | 35.242 (33.323,37.161) | 3.978 (3.838,4.117) | 3.917 (3.772,4.061) | 4.053 (3.871,4.234) |
| 2032 | 44.813 (41.749,47.878) | 48.899 (43.326,54.473) | 35.581 (33.515,37.646) | 4.012 (3.865,4.158) | 3.95 (3.799,4.102) | 4.087 (3.896,4.278) |
| 2033 | 45.238 (42,48.477) | 48.905 (42.733,55.078) | 35.92 (33.714,38.125) | 4.046 (3.892,4.199) | 3.984 (3.825,4.143) | 4.121 (3.921,4.322) |
| 2034 | 45.663 (42.26,49.067) | 48.828 (42.043,55.614) | 36.259 (33.921,38.598) | 4.08 (3.92,4.24) | 4.018 (3.853,4.184) | 4.156 (3.946,4.365) |
| 2035 | 46.089 (42.528,49.649) | 48.713 (41.29,56.136) | 36.6 (34.134,39.065) | 4.114 (3.947,4.28) | 4.052 (3.88,4.224) | 4.19 (3.972,4.408) |
| 2036 | 46.514 (42.802,50.225) | 48.629 (40.548,56.709) | 36.94 (34.352,39.527) | 4.148 (3.975,4.32) | 4.086 (3.908,4.264) | 4.224 (3.998,4.45) |
